# Evaluation the protective role of baicalin against H_2_O_2_-driven oxidation, inflammation and apoptosis in bovine mammary epithelial cells

**DOI:** 10.3389/fvets.2024.1504887

**Published:** 2024-12-12

**Authors:** Xiaohui Kong, Mingyan Wang, Zhiheng Guo, Xingda Yang, Hongxia Lian, Tengyun Gao, Liyang Zhang, Tong Fu

**Affiliations:** ^1^Henan International Joint Laboratory of Nutrition Regulation and Ecological Raising of Domestic Animal, College of Animal Science and Technology, Henan Agricultural University, Zhengzhou, China; ^2^Department of Economic Management and Animal Husbandry, Ruzhou Vocational and Technical College, Pingdingshan, China

**Keywords:** baicalin, bovine mammary epithelial cells (BMECs), nuclear factor erythroid 2-related factor 2 (Nrf2), inflammation, oxidative stress, apoptosis

## Abstract

Mastitis is one of the most common diseases in dairy farms. During the perinatal period, the bovine mammary epithelial cells (BMECs) of High-yielding dairy cows accelerate metabolism and produce large amounts of reactive oxygen species (ROS). It is one of the primary causes of mastitis and will lead to the breakdown of redox balance, which will induce oxidative stress, inflammation, and apoptosis. Baicalin is a flavonoid substance extracted from the root of natural plant *Scutellaria baicalensis*, which has anti-inflammatory, anti-oxidant, anti-viral and other biological functions. In this research, hydrogen peroxide (H_2_O_2_) was used to construct a mastitis oxidative stress model, and relevant mechanisms were analyzed by immunofluorescence techniques, qRT-PCR and Western Blot to explore how baicalin affects BMECs' oxidative stress and inflammation caused by H_2_O_2_, as well as to provide new perspectives on the combined application of baicalin in the prevention and treatment of mastitis. The results demonstrated that baicalin treatment could reduce the accumulation of H_2_O_2_-induced intracellular ROS and decrease the expression of inflammatory cytokines Tumor Necrosis Factor-α (TNF-α), interleukin 6 (IL-6), interleukin-1β (IL-1β) and the apoptosis rate. The inhibitory effect of baicalin on H_2_O_2_-induced intracellular ROS accumulation and the expression of inflammatory cytokines and apoptotic factors in BMECs was blocked by pretreatment with the Nuclear factor erythroid 2-related factor 2 (Nrf2) inhibitor retinoic acid (RA) prior to H_2_O_2_ and/or baicalin treatment. In summary, baicalin could served as a natural antioxidant agent to regulate cell apoptosis through its anti-inflammatory, antioxidant and anti-apoptotic effects to combat BMECs damage caused by H_2_O_2_.

## Introduction

Mastitis is one of the main diseases in dairy cows, which not only affects their health but also reduces milk production and quality. Especially during the perinatal period and peak lactation period, the extensive synthesis of breast milk raises the energy requirements of breast tissue. When the synthesis rate of reactive oxygen species (ROS) is greater than the removal rate of antioxidants, this will cause excessive accumulation of ROS in the body, destroying cell membrane structure and resulting in oxidative stress (1, 2). The excessive accumulation of ROS could also destroy the dynamic balance of the antioxidant defense system, induce cell apoptosis, and increase the incidence of mastitis (3). Hydrogen peroxide (H_2_O_2_) is an inorganic oxide. Numerous studies have demonstrated that H_2_O_2_ could induce the overproduction of ROS and initiate oxidative stress through a feedback system involving intricate physiological processes, such as apoptosis and autophagy, and H_2_O_2_-treated BMECs could more accurately simulate the actual state of oxidative damage and mastitis in dairy cows, with stable properties (4–6). Therefore, the H_2_O_2_ was used in the current study to construct an oxidative stress model, providing a basis for investigating the potential mechanism and effective intervention measures of cell apoptosis.

The bovine mammary gland epithelial cells (BMECs) are the primary cellular components of the cow mammary gland, not only synthesizing and secreting milk but also playing an important role in the innate immunity of the gland (7). Metabolic activity is enhanced during lactation, and BMECs are highly susceptible to oxidative stress in breast tissue (8). Therefore, it is becoming increasingly important to take measures to reduce oxidative stress damage to BMECs in dairy cows. The nuclear factor erythroid 2-related factor 2 (Nrf2) is a major transcriptional protein regulating oxidative damage. When cells are under oxidative stress, Nrf2 dissociates from Keap1, transported to the nucleus to bind to ARE, activating the expression of downstream antioxidant genes, such as quinone oxidoreductase 1 (NQO1) and heme oxygenase 1 (HO-1) (9, 10). An essential inflammatory signaling pathway, activation of nuclear factor kappa-B (NF-κB), could increase the synthesis of pro-inflammatory proteins such as Tumor Necrosis Factor-α (TNF-α), interleukin 6 (IL-6), and interleukin-1β (IL-β). It has been reported that Nrf2 activation reduces the incidence of inflammatory reactions by inhibiting the expression of IκB (inhibitor of NF-κB) phosphorylation and the NF-κB pathway. However, this effect is eliminated when Nrf2 and HO-1 are inhibited, indicating that Nrf2 signaling plays an indispensable role in NF-κB-mediated inflammation (11, 12). In addition, excessive ROS can cause irreversible cell damage, such as degradation of cell structure and components, as well as DNA damage, leading to cell apoptosis. In addition, excessive ROS could cause irreversible cellular damage, such as degradation of cell structure and components and DNA damage, which leads to apoptosis (13). Therefore, alleviation of oxidative stress is crucial to inhibit inflammation and apoptosis in BMECs, and Nrf2 might be a potential therapeutic target for the reduction of oxidative stress.

Baicalin is one of the flavonoids extracted from the dried root of the herb *Scutellaria baicalensis*, exhibiting antibacterial, anti-inflammatory, antioxidant and anti-tumor properties. Numerous studies have demonstrated that baicalin could reduce malondialdehyde (MDA) content and increase the activity of superoxide dismutase (SOD) and total antioxidant capacity (T-AOC) through the Nrf2 pathway, thereby exerting antioxidant effects (14, 15). By suppressing NF-κB and its downstream inflammatory cytokines, as well as the caspase3 signaling pathways, baicalin could reduce oxidative stress by scavenging ROS and controlling inflammation and apoptosis (16, 17). However, there is still a lack of in-depth systematic studies on whether the effects of baicalin on inflammation and apoptosis are mediated by oxidative stress and whether the above changes also occur in BMECs. Therefore, the purpose of this study was to investigate the effect and mechanism of baicalin on H_2_O_2_-induced oxidative stress and apoptosis of BMECs.

## Materials and methods

### Cell culture

The BMECs were obtained from the Physiological and Biochemical Laboratory of Henan Agricultural University and retained the characteristics and functions of the primary cells. Add 10% fetal bovine serum (FBS; Gibco, New York, USA) at 37°C and culture using 5% CO_2_ and 1% penicillin/streptomycin/amphotericin B sterile solution (Solarbio, Beijing, China). After 48 h of initial culture, the cells were cleaned with phosphate-buffered saline (PBS; BI, Israel) and the culture medium was removed. Then, the cells were digested with 0.25% trypsin-ethylenediamine tetraacetic acid (Gibco, New York, USA) for 3 min and stop digestion by adding DMEM/F12 containing 10% FBS. Centrifuge the cells for 4 min, then reculture in the medium and incubate at 37°C in a 5% CO_2_ incubator at 37°C. The cells were used for follow-up experiments when the cell coverage reached 80%.

### Sample treatment

Baicalin (C_21_H_18_O_11_, purity > 98%, Macklin Biochemical Co., Ltd, Shanghai, China), hydrogen peroxide (H_2_O_2_, purity 3%, Merck & Co Inc, New Jersey, USA), and retinoic acid [RA, specific inhibitor of Nrf2 (Proteintech, Chicago, IL), Med Chem Express, New Jersey, USA] were stored at room temperature. Baicalin was fully dissolved in dimethyl sulfoxide (DMSO; Solarbio, Beijing, China) to the final concentration of 500 μM and stored at 4°C protected from light. Four experimental groups were set up in order to investigate whether reducing oxidative stress could decrease the inflammatory response, including control group (CON, untreated), H_2_O_2_ treatment group (H_2_O_2_), baicalin treatment group (baicalin) and baicalin with H_2_O_2_ group (baicalin +H_2_O_2_), baicalin and H_2_O_2_ group cells were finally treated with 10 and 200 μM for 18 and 6 h, and baicalin with H_2_O_2_ group cells were pretreated with 10 μM dose for 18 h. Then, the cells were treated with 200 μM H_2_O_2_ for 6 h. The baicalin solution was diluted with cell culture medium to 5, 10, 25, 50, and 100 μM for 12, 24, and 48 h to explore the alleviating effect of baicalin concentration on H_2_O_2_-induced oxidative stress, respectively. The same volume of DMSO was added to the CON group, ensuring that the concentration of DMSO in all treatment solutions prepared was < 0.1% (v/v). The H_2_O_2_ was diluted with cell culture medium to concentrations of 100, 200, 400, 600, 800, and 1,000 μM, and treat the cells for 4, 6, and 8 h, respectively. According to the results, treating cells with 10 μM baicalin for 24 h and inducing cells with 200 μM H_2_O_2_ for 6 h were used as the optimal treatment conditions.

### Cell viability assay

The appropriate H_2_O_2_ concentration and exposure time were screened for treating cells with Cell Counting kit-8 (CCK-8, Beijing Sun Biotechnology Co., LTD). The BMECs (2 × 10^5^ cells/mL) were inoculated into 96-well plates, and the culture medium was discarded when the cells proliferated to the density of 70–80%. Then, 100 μL of different concentrations of baicalin and/or H_2_O_2_ solution was added to the wells and incubated at 37°C in a 5% CO_2_ incubator for an appropriate time. A serum-free cell culture medium was used to wash the cells twice. Then, 100 μL 10% CCK-8 solution was added to each well. After incubation in the incubator for 2 h, the absorbance values at 450 nm were measured using an enzyme marker (BioTek Instruments, Inc, USA). Cell viability was calculated as follows: (OD of treatment group-OD of the blank group)/(OD of control group-OD of the blank group) × 100%.

### ROS determination

The intracellular ROS were detected by fluorescent probe DCFH-DA staining using Reactive Oxygen Species Assay Ki (ROS Assay Kit, Shanghai Beyotime Biotechnology Co., Ltd. Shanghai, China). The cells were washed with PBS and then coincubated with 10 μM DCFH-DA in a cell incubator for 20 min. The cells were fully washed with PBS to remove DCFH-DA that had not entered the cells. Finally, the cells were resuspended in PBS and immediately visualized and imaged using fluorescence microscopy (AmScope, California, USA). After the same procedure, the fluorescence intensity at 488 nm excitation wavelength and 525 nm emission wavelength was measured using a Multifunctional Enzyme Labeler (SpectraMax i3x, Silicon Valley, USA).

### Oxidative damage indicators measurement

Collecting the cells and lysing with RIPA lysis buffer (Beyotime, Shanghai, China) containing 1% phosphatase inhibitor mixture (CWBIO, Beijing, China) and protease inhibitor cocktail (Epizyme, Shanghai, China). The supernatant was removed after centrifugation, and the protein concentrations of samples were determined using a BCA kit (Beyotime, Shanghai, China). Then, the MDA content, SOD activity, and T-AOC level in the protein sample were measured according to the instructions of the Lipid MDA Assay Kit (S0131M), Total Superoxide Dismutase Assay Kit (S0101S), and Total Antioxidant Capacity Assay Kit (WST-8) (Beyotime Biotechnology Co. Shanghai, China).

### Quantitative real-time PCR assay

Total RNA was extracted from BMECs by TRIzol reagent (Quan-Style Gold, Beijing, China), and the quality of RNA was detected. Then, 1 μg of total RNA samples was reverse-transcribed to cDNA according to the operation instructions (Vazyme, Nanjing, China), and the relative expression of mRNA was detected using a 7500 Real-Time PCR system (Applied Biosystems). The reactions were as follows: predenaturation (95°C for 30 s); cyclic reaction 40 times (95°C for 5 s, 60°C for 30 s); melting curve (95°C for 5 s, 65°C for 5 s). Three biological replicates and three technical replicates were set up for each treatment group to determine the mean crossover point value and standard deviation (SD). The fluorescence quantification results were calculated using the 2-ΔΔCT method, and the results were normalized using β-actin as an internal reference gene. The primers were designed by NCBI and synthesized by Shangya Biologicals. The sequences of the synthesized primers are shown in Table 1.

**Table 1:** Primer sequences of the genes.

**Gene**	**Primer sequences(5^′^-3^′^)**	**Length (bp)**
NQO1	F GATCGTACTGGCCCACTCAG	169
	R GGGGTCCTTCAGTTTACCTGT	
HO-1	F CATCGACCCCACACCTACAC	192
	R AAGACGCCATCACCAGCTTA	
IL-6	F ATCTGGGTTCAATCAGGCGAT	200
	R CAGTGTTTGTGGCTGGAGTG	
IL-1β	F CCCCAGAGGGAAGAGCAGT	168
	R GAGGGCATTGGCATACGAGT	
TNF-α	F GAGCCTGTGAGCGTGCTTTT	163
	R TGGTGCTGAGGATGACATGG	
β-actin	F GATGATGATATTGCTGCGCTCG	159
	R TACGAGTCCTTCTGGCCCAT	

### Western blot analysis

Proteins were extracted from BMECs using RIPA lysis buffers containing protease and phosphatase inhibitors, and their concentrations were determined. The protein samples were mixed with 5 × SDS–PAGE Protein Sample Loading Buffer (Beyotime, Shanghai, China) proportionally and then heated together in a water bath at 98°C for 5 min to denature the proteins. Separation and concentration gels were prepared according to the molecular weight requirement of the target protein, and the denatured protein samples and marker were added to the lane in turn. Subsequent to electrophoresis, the protein bands were transferred to polyvinylidene fluoride (PVDF) membranes according to the wet transfer method. The PVDF membranes were blocked in a shaker with 5% skimmed milk for 1 h before incubation with the appropriate primary antibody overnight at 4°C. The next day, PVDF membranes were first washed 3 times with Tris Buffered Saline with Tween 20 (TBST) for 15 min each. Then, they were incubated for 1 h at room temperature using the corresponding matching fluorescent-coupled secondary antibody and washed again with TBST. The protein bands were visualized by Odyssey Infrared Imaging (LI-COR Biosciences, USA) using the Omni-ECL™ Femto Light Chemiluminescence Kit (Epizyme, Shanghai, China). The target proteins expression levels were normalized using β-actin bands as an internal control, and the protein bands were quantitatively analyzed by ImageJ software.

### Statistical analysis

Data were collated and plotted by GraphPad Prism 8.0 software (San Diego, CA). One-way ANOVA and Duncan's multiple range test were used for multiple comparisons. The results are expressed as the means ± standard error (SEM) with three replicates for each treatment, and *P* < 0.05 indicated a significant difference.

## Results

### Protective effect of baicalin against H_2_O_2_-induced cell damage

**Figure 1:**
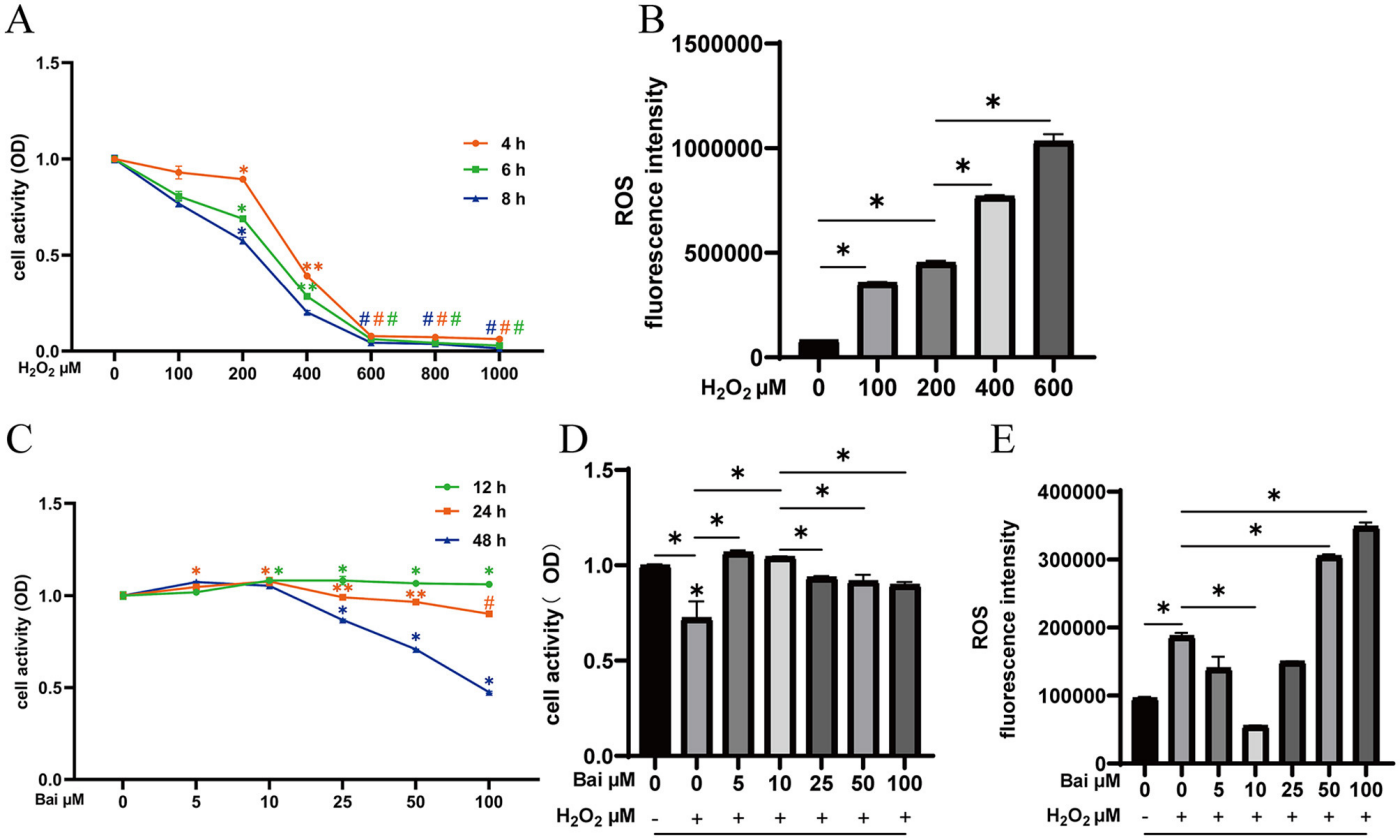
Exploration of the optimal concentration and time of H_2_O_2_ and baicalin. **(A)** Effect of different concentrations of H_2_O_2_ (0, 100, 200, 400, 600, 800, and 1,000 μM) on the cell viability of BMECs upon stimulation for 4, 6, and 8 h. *P < 0.05 compared with the CON, **P < 0.05 compared with the 200 μM H_2_O_2_ group, ^#^*P* < 0.05 compared with the 400 μM H_2_O_2_ group. **(B)** Effect of different concentrations of H_2_O_2_ on ROS levels in BMECs upon stimulation for 6 h. *P < 0.05. **(C)** Effect of different concentrations of baicalin (0, 5, 10, 25, 50, 100 μM) on the cell viability of BMECs upon stimulation for 12, 24 and 48 h. *P < 0.05 compared with the CON, **P < 0.05 compared with the 10 μM baicalin group, ^#^*P* < 0.05 compared with the 50 μM baicalin group. **(D)** BMECs were treated with baicalin at different concentrations, and 200 μM H_2_O_2_ was added 6 h before the end of treatment to determine the cell viability at 24 h. *P < 0.05. **(E)** Effect of different concentrations of baicalin on H_2_O_2_-induced ROS levels in BMECs. *P < 0.05. The data from the CON were used to normalize the data of each treatment group. Each treatment was repeated three times, and the results are expressed as the means ± SEM.

Results of the protective effect of baicalin on H_2_O_2_-induced cell damage are shown in Figure 1. The cell viability of BMECs decreased to 68.9% after 6 h of stimulation with 200 μM H_2_O_2_, which the results satisfying the experimental requirements for establishing an oxidative damage model (Figure 1A). The treatment of 200 μM H_2_O_2_ significantly increased cells' ROS levels compared to the CON (*P* < 0.05) (Figure 1B). Different concentrations of baicalin were used to stimulate the cells for 12, 24 and 48 h, with varying effects (*P* < 0.05) (Figure 1C). Under the above conditions, the concentration of baicalin at 10 μM and the treatment time of 24 h could effectively inhibit H_2_O_2_-induced ROS level increase (*P* < 0.05) (Figures 1D, E).

### Baicalin alleviates H_2_O_2_-induced oxidative stress

**Figure 2:**
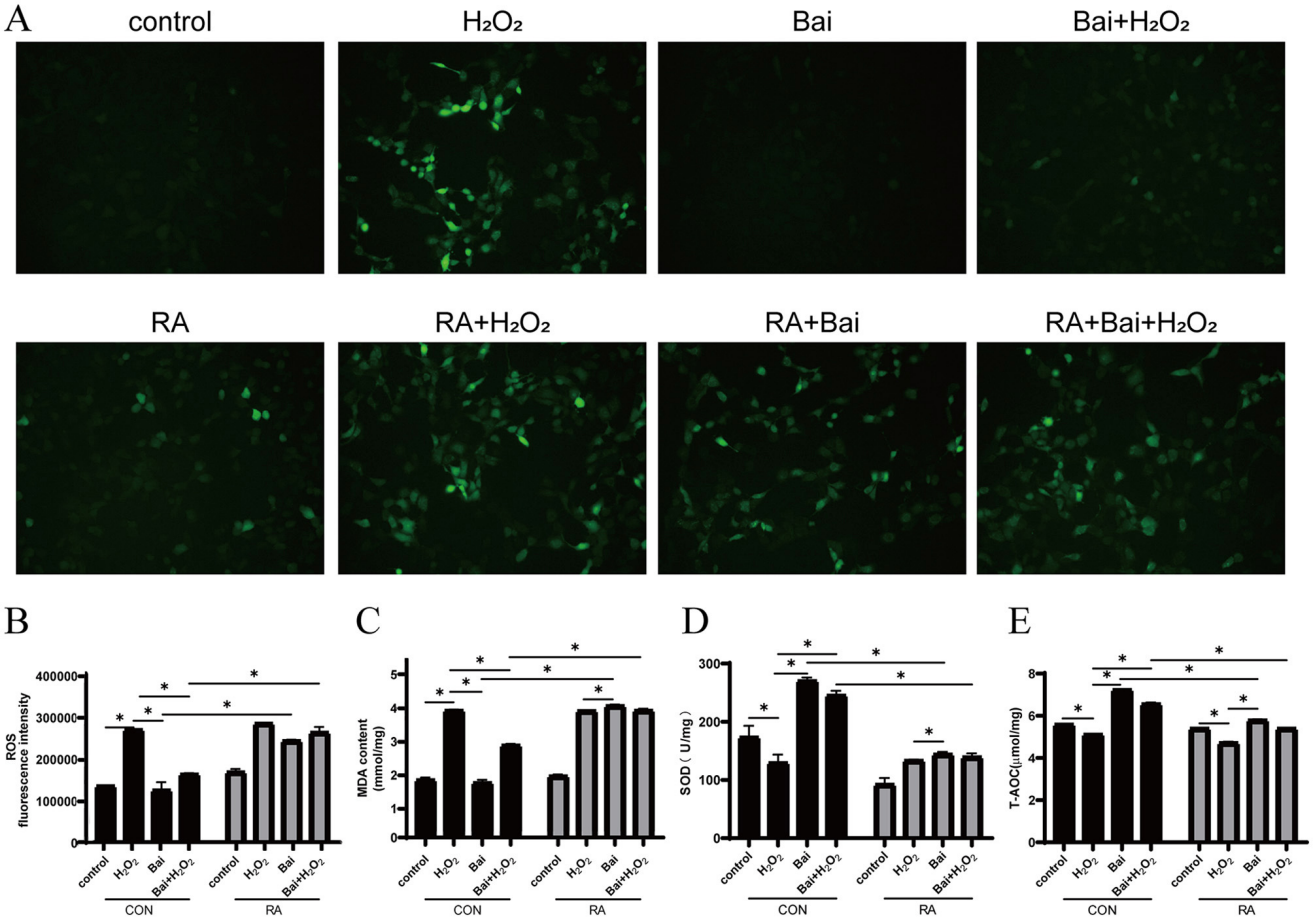
Effect of baicalin on H_2_O_2_-induced changes in ROS, MDA, SOD and T-AOC in BMECs. BMECs were treated with 5 μM RA for 1 h, and baicalin (10 μM) was then added for 18 h before cotreatment with 200 μM H_2_O_2_ for 6 h. **(A)** The ROS fluorescence intensity was imaged using a fluorescence microscope (scale bar is 200 μm). **(B)** Changes in ROS levels in BMECs. **(C)** Changes in MDA content in BMECs. **(D)** Changes in SOD activity in BMECs. **(E)** Changes in T-AOC levels in BMECs. *P < 0.05. RA, Nrf2 inhibitor.

Next, it explored whether the antioxidant effect of baicalin was related to Nrf2 by adding the Nrf2 inhibitor RA. As shown in Figure 2, compared with the CON group, the H_2_O_2_ treatment increased ROS levels (*P* < 0.05) (Figures 2A, B) and MDA content in cells (*P* < 0.05) (Figure 2C), decreased SOD activity (*P* < 0.05) (Figure 2D) and T-AOC levels (*P* < 0.05) (Figure 2E). Furthermore, after the addition of Nrf2-specific inhibitor RA, baicalin was unable to effectively inhibit the increase of ROS and MDA levels (*P* < 0.05) (Figures 2A–C), and the levels of SOD and T-AOC were significantly decreased comparison to the CON group (*P* < 0.05) (Figures 2D, E).

### Baicalin activates Keap1/Nrf2 signaling pathway

**Figure 3:**
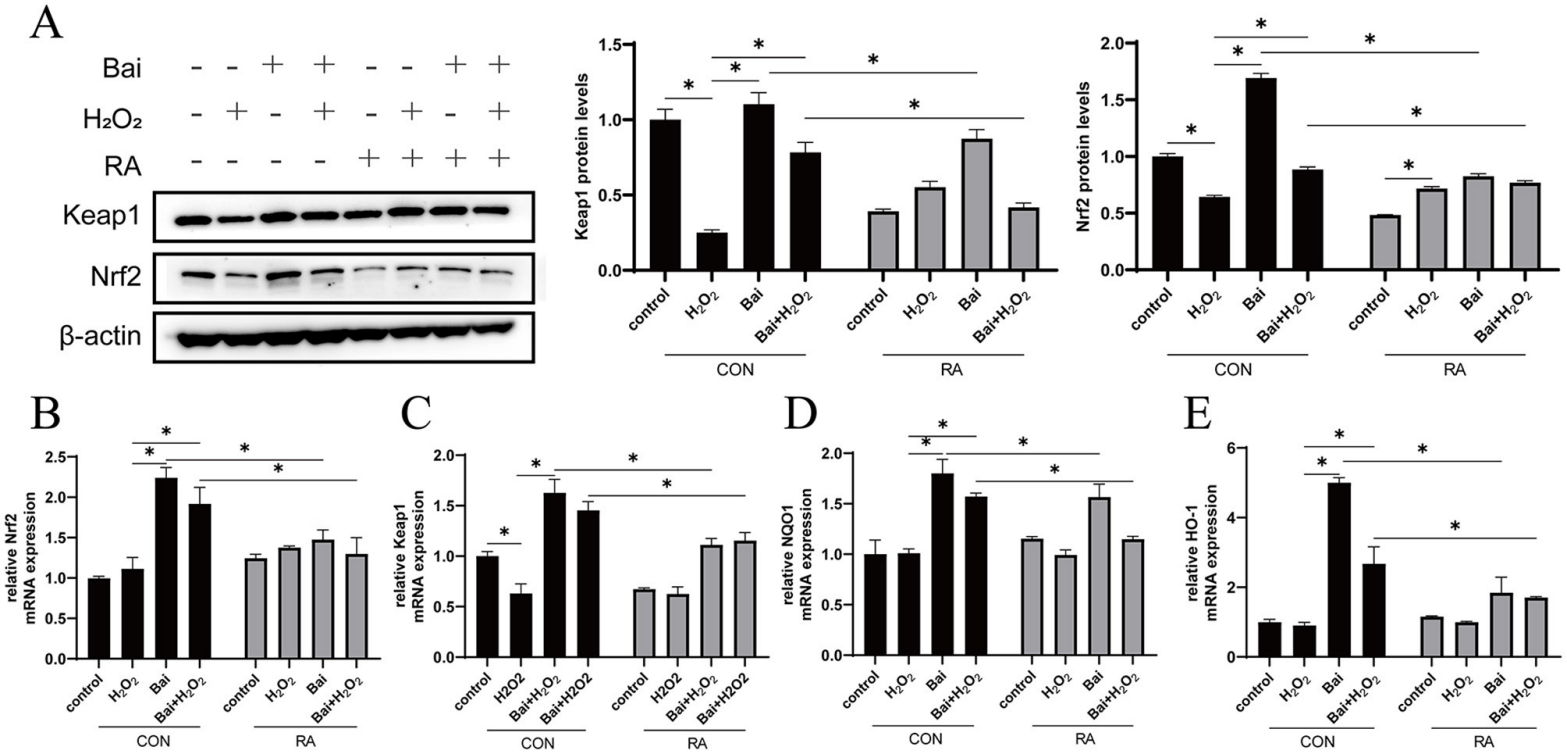
Activation of the Keap1/Nrf2 signaling pathway by baicalin. BMECs were treated with 5 μM RA for 1 h, and baicalin (10 μM) was then added for 18 h before cotreatment with 200 μM H_2_O_2_ for 6 h. **(A)** Western blot analysis of Keap1 and Nrf2 protein levels in BMECs. Immunoreactive bands are shown on the left, and quantitative results are shown on the right. **(B)** Nrf2 mRNA levels in BMECs. **(C)** Keap1 mRNA levels in BMECs. **(D)** HO-1 mRNA levels in BMECs. **(E**) NQO1 mRNA levels in BMECs. *P < 0.05.

The H_2_O_2_ treatment significantly reduced Nrf2 and Keap1 protein expression levels (*P* < 0.05) compared to the CON group (Figure 3A). Then, the above results were further verified by qRT-PCR, and the expression of Nrf2 and Keap1 mRNA was consistent with the Western blotting results (*P* < 0.05) (Figures 3B, C). In addition, baicalin treatment also increased the mRNA abundance of the downstream antioxidant genes NQO1 and HO-1, while the addition of RA inhibited the activation of NQO1 and HO-1 by baicalin (*P* < 0.05) (Figures 3D, E).

### Baicalin inhibits the H_2_O_2_-induced NF-κB signaling pathway

**Figure 4:**
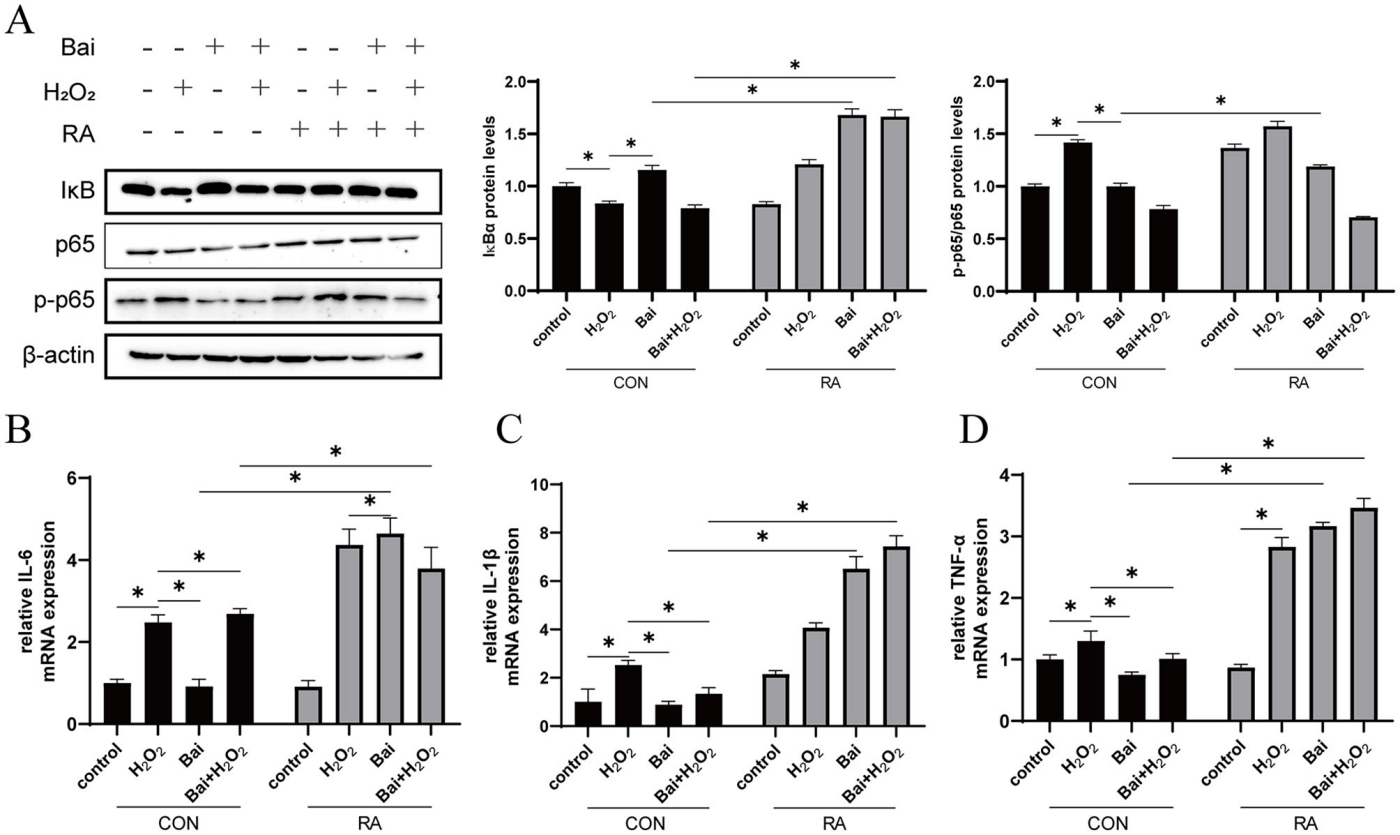
Baicalin inhibits the H_2_O_2_-induced NF-κB signaling pathway. BMECs were treated with 5 μM RA for 1 h, and baicalin (10 μM) was then added for 18 h before cotreatment with 200 μM H_2_O_2_ for 6 h. **(A)** Western blot analysis of IκBα, p65 and p-p65 protein levels in BMECs. Immunoreactive bands are shown on the left, and quantitative results are shown on the right. **(B)** IL-6 mRNA levels in BMECs. **(C)** IL-1β mRNA levels in BMECs. **(D)** TNF-α mRNA levels in BMECs. *P < 0.05.

The effects of baicalin on BMECs inflammation and the regulatory effects of Nrf2 on inflammation are shown in Figure 4. Compared with the CON group, H_2_O_2_ treatment decreased the IκBα protein expression level and increased the p-p65/p65 ratio (*P* < 0.05) (Figure 4A), while baicalin had the opposite effect. This effect of baicalin was reversed after the addition of RA. Interleukin IL-6, IL-1β, and tumor necrosis factor TNF-α are the main cellular inflammatory factors and play an important role in BMECs inflammatory response. H_2_O_2_ treatment significantly increased the mRNA content of the downstream pro-inflammatory cytokines IL-1β, IL-6, and TNF-α (*P* < 0.05) (Figures 4B–D). However, with or without cotreatment with H_2_O_2_, baicalin inhibited the mRNA expression of IL-1β, IL-6, and TNF-α, and the addition of RA effectively blocked the inhibitory effect of baicalin.

### Baicalin inhibits the H_2_O_2_-induced caspase-3/Bcl-2 signaling pathway

**Figure 5:**
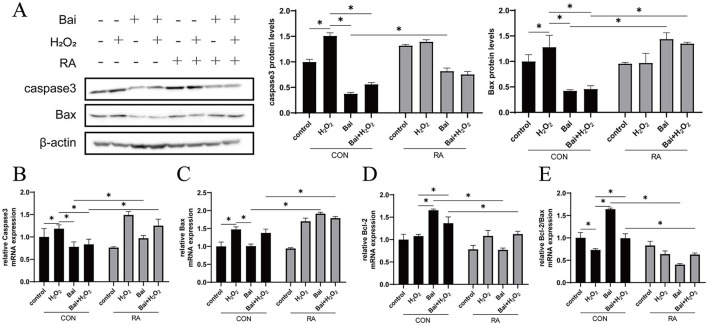
Baicalin inhibits the H_2_O_2_-induced caspase3/Bcl-2 signaling pathway. BMECs were treated with 5 μM RA for 1 h, and baicalin (10 μM) was then added for 18 h before cotreatment with 200 μM H_2_O_2_ for 6 h. **(A)** Western blot analysis of caspase3 and Bax protein levels in BMECs. Immunoreactive bands are shown on the left, and quantitative results are shown on the right. **(B)** Caspase3 mRNA levels in BMECs. **(C)** Bax mRNA levels in BMECs. **(D)** Bcl-2 mRNA levels in BMECs. **(E)** Ratio of Bcl-2 to Bax mRNA levels in BMECs. *P < 0.05.

The H_2_O_2_ treatment increased caspase-3 and Bax protein expression levels (*P* < 0.05) (Figure 5A), while baicalin treatment had an opposite effect on H_2_O_2_ with or without cotreatment with H_2_O_2_. This effect of baicalin was inhibited by the addition of RA. In addition, the above results were further validated using qRT–PCR, which showed that H_2_O_2_ treatment increased caspase-3 and Bax mRNA abundance and decreased the Bcl-2/Bax ratio (*P* < 0.05) (Figures 5B–E). However, baicalin treatment decreased caspase-3 and Bax mRNA abundance and increased Bcl-2 and Bcl-2/Bax ratios (*P* < 0.05), and the addition of RA effectively inhibited the effect of baicalin.

## Discussion

The BMECs play a crucial role in the antioxidant process as the first line of defense against the invasion of pathogenic microorganisms and are an ideal model for establishing oxidative stress *in vitro*. In the current study, BMECs induced by H_2_O_2_ were used to construct an oxidative stress model. The results revealed that the induction of BMECs with 200 μM H_2_O_2_ for 6 h drastically decreased cell viability as well as increased ROS levels. In contrast, treating BMECs with 10 M baicalin for 24 h reversed the damage caused by H_2_O_2_ to BMECs. The biological effect of baicalin in enhancing cell growth might be the cause of these outcomes. Perruchot et al. demonstrated that baicalin treatment of BMECs could inhibit the synthesis of ROS and free radicals, providing a protective function in the body's antioxidant defense system (18). It illustrated that low-concentration baicalin (1–10 μg/mL) pre-treatment protected BMECs from H_2_O_2_-induced oxidative stress damage by regulating cell proliferation and the antioxidant response.

All organisms could produce ROS during cellular metabolism (19, 20). Numerous biological processes, such as oxidative stress, inflammation, and cell death, are linked to ROS. However, the breakdown of antioxidant defense systems leads to a decrease in antioxidant capacity and an overabundance of ROS, which triggers oxidative damage (20, 21). Relevant studies have shown that flavonoids, the primary active ingredient in baicalin, could effectively prevent peroxide and inhibit the occurrence of free radical chain reactions (14, 15). In this study, baicalin was found to reduce the accumulation of ROS in H_2_O_2_-induced BMECs. These results suggest that baicalin plays an antioxidant role in reducing H_2_O_2_-induced ROS accumulation. The MDA content and SOD activity in cells could reflect the degree of free radical damage (21, 22). The current research results have found that H_2_O_2_ increased the MDA content in BMECs, while baicalin treatment significantly reduced the MDA content in cells, increased SOD activity and T-AOC levels. The results are consistent with a recent study by Wang et al. (22) and Zhou et al. (23), which showed that baicalin supplementation significantly reduced the levels of ROS and MDA. It is indicated that baicalin could enhance the antioxidant defense system and protect cells from oxidative stress and apoptosis. After Nrf2 is activated, it could regulate the gene expression of antioxidant-related enzymes and proteins (24). Therefore, the antioxidant effect of baicalin might be related to the Nrf2 signaling pathway. To further verify this, we added the Nrf2 inhibitor RA and found that RA effectively blocked the antioxidant effect of baicalin. Therefore, current research confirmed that baicalin has the potential to prevent oxidative stress and might mediate the action of Nrf2.

The Nrf2 pathway is a key regulator in the cellular anti-oxidative stress and defense system (25), and its expression could be activating and negatively regulated through the N-terminal structural domain by Kelch, like ECH-related protein 1 (26). Therefore, to verify the effect of baicalin on Nrf2, the expression of Nrf2 and its related proteins was examined in this study. The results showed that Nrf2 was activated by Keap1 degradation, which resulted in the subsequent translocation in the presence or absence of H_2_O_2_. Baicalin treatment significantly increased the expression levels of Nrf2, Keap1, NQO1 and HO-1 proteins, and the addition of RA inhibited the promotion of Nrf2 and Keap1 protein expression by baicalin. Ma et al. also discovered that baicalin could play an antioxidant role by activating the Nrf2-mediated antioxidant pathway and increasing the expression of HO-1, which is consistent with the conclusion of this study (27). Previous studies have shown that baicalin could prevent Keap1 from binding to Nrf2 through a modified Keap1 protein complex, resulting in reduced ubiquitination and thereby encouraging the expression of Nrf2 (28). These results demonstrated that baicalin could maintain the dynamic homeostasis of the body by activating the Keap1/Nrf2 signaling pathway, thus protecting BMECs from H_2_O_2_-induced oxidative stress.

It has been reported that baicalin could effectively inhibit allergic inflammation exudation, reduce capillary permeability, and inhibit intestinal inflammation (29). In this study, H_2_O_2_ treatment increased the mRNA levels of downstream pro-inflammatory cytokines IL-6, IL-1 β, and TNF-α, and this effect was reversed by baicalin supplementation. Shen reported that baicalin pretreatment could reduce the levels of IL-6, IL-1 β, and TNF-α in a mouse lung inflammation model, thus improving pathological changes in the lungs and reducing the body's inflammatory reaction (30). Consequently, the mechanism of anti-inflammatory baicalin might involve the inhibition of inflammation-related proteins and the blocking of NF-κB pathway activation (31). During the resting state, the NF-κB/Rel transcription factor binds to the upstream inhibitory κB kinase (IκB) to form a complex and is present in the cytoplasm in an inactive form. When cells are triggered, IκB kinase β (IKKβ) is phosphorylated, leading to the hydrolysis of IκBα by proteases and the release of NF-κB/Rel transcription factors into the nucleus as NF-κB p65, which in turn regulates the expression of downstream pro-inflammatory cytokines. ROS could directly activate IκB and induce nuclear translocation of the inflammatory mediator NF-κB, thereby regulating the expression of inflammation- and oxidative stress-related genes (32, 33). Interestingly, many of the downstream products encoded by NF-κB are in turn activators of ROS, thus producing feedback effects exacerbating oxidative stress and ultimately creating a vicious cycle (34, 35). Therefore, we investigated the effect of baicalin on NF-κB related genes. The results indicated that baicalin could reduce the expression of NF-κB. Zhang et al. (36) and Li et al. (37) showed that baicalin protects cells from inflammatory damage by down-regulating the expression of the transcription factor NF-κB. Jiang reported that baicalin pretreatment could reduce the levels of IL-6, IL-1β and TNF-α in the cell supernatant, thereby significantly reducing the activity of NF-κB and reducing the inflammatory reaction of the body (38). It is consistent with the results of this study. Meanwhile, it has been discovered that activation of Nrf2 could inhibit the NF-κB inflammatory signaling pathway, reduce DNA oxidative damage, and thus alleviate inflammation and apoptosis (2). To further verify whether Nrf2 regulates the inflammatory response, we added the Nrf2 inhibitor RA and found that it blocked the inhibitory effect of baicalin on inflammatory cytokines, such as NF-κB. This result confirmed that Nrf2 mediates the regulatory effect of baicalin on inflammation.

Furthermore, apoptosis is also mediated by oxidative stress (39). We found that the modulation of oxidative stress by baicalin might inhibit apoptosis. Caspases are usually present as non-activated zymogens in most animal cells, and caspase zymogen activation is a central process in the onset of apoptosis, where caspase-3 is mainly involved in the execution of apoptosis. The apoptosis-related genes Bcl-2 and Bax belong to the Bcl-2 family. Bax could promote apoptosis, while Bcl-2 is an anti-apoptotic protein that could inhibit the activation of caspase to prevent apoptosis (40). It has been shown that activation of the Nrf2 signaling pathway could effectively alleviate mitochondrial damage and inhibit apoptosis (2, 41). Therefore, we explored the effect of Nrf2 signaling pathway inhibition on apoptosis. Baicalin significantly inhibited the H_2_O_2_-induced expression of caspase-3 and Bax and upregulated the expression of Bcl-2, while the inhibitor RA reversed these effects. Previous studies have shown that when apoptotic signals stimulate cells, the pro-apoptotic protein Bax oligomerizes and induces an increase in mitochondrial membrane permeability. It subsequently promotes the release of cytochrome c from the mitochondria, activates downstream caspase-3, and initiates the caspase cascade, resulting in the specific cleavage of downstream substrates, thereby triggering apoptosis. It is reported that baicalin supplementation could increase cell viability, elevate mitochondrial membrane potential, upgrade Bcl-2 levels and decrease caspase-3 and Bax levels, and our study also confirms this finding (42). This result suggests that baicalin might regulate the caspase/Bcl-2 signaling pathway through Nrf2 mediation, thereby inhibiting apoptosis.

## Conclusions

**Figure 6:**
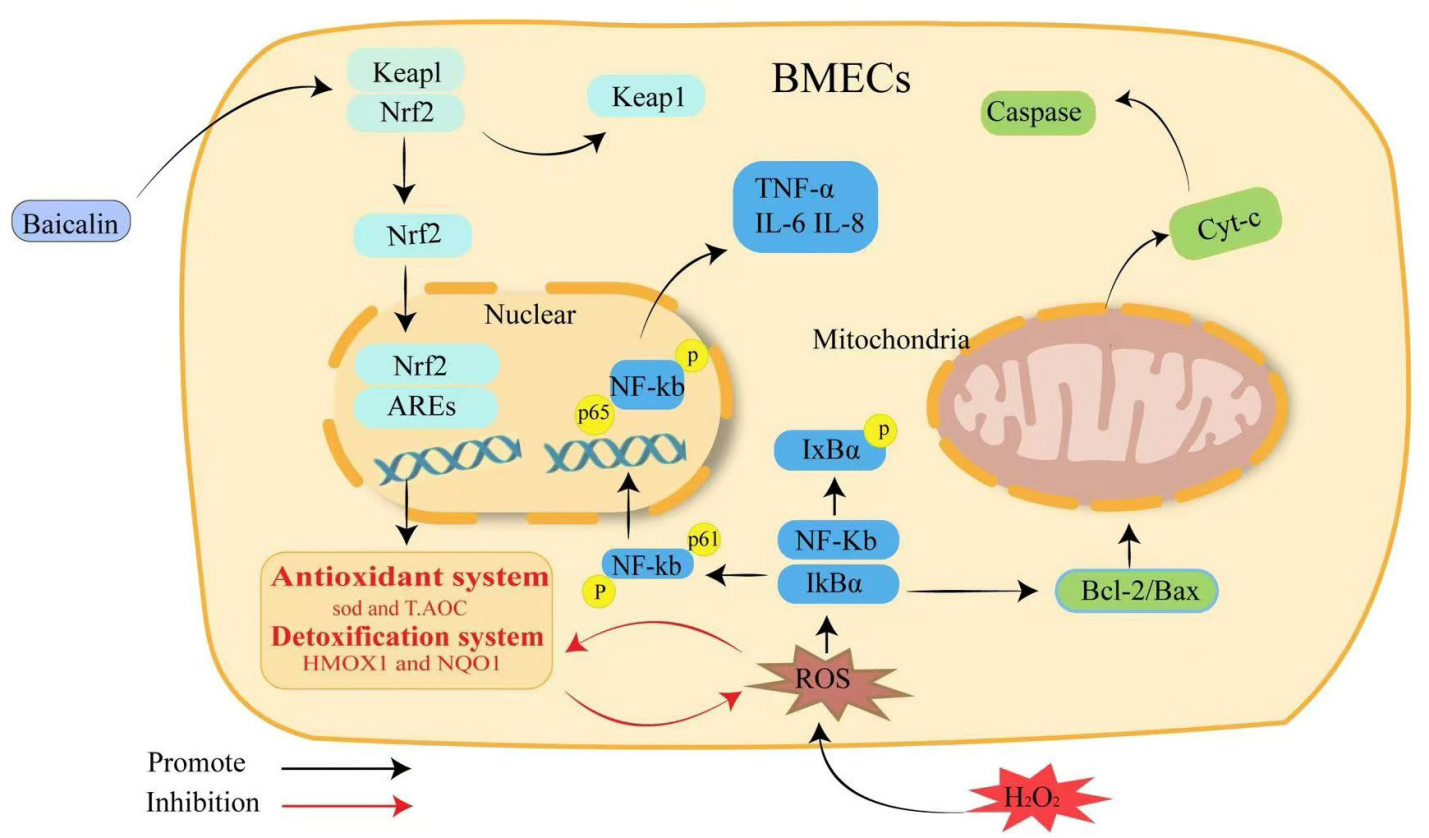
Schematic summary of the effect and mechanism of baicalin on H_2_O_2_-induced oxidative stress, inflammation and apoptosis in BMECs. Baicalin resists H_2_O_2_-induced oxidative damage in BMECs by activating the Keap1/Nrf2 signaling pathway and reducing ROS production, which in turn inhibits NF-κB inflammation and the caspase3/Bcl-2 apoptotic pathway to maintain intracellular redox homeostasis.

In conclusion, BMECs are stimulated by H_2_O_2_, inducing oxidative damage and inflammation, disrupting cellular homeostasis, and leading to cell apoptosis. Baicalin could increase the expression of downstream antioxidant genes NQO1 and HO-1, as well as antioxidant system SOD and T-AOC, by activating Keap1/Nrf2 signaling pathway, thus alleviating H_2_O_2_-induced oxidative damage in BMECs. The NF-kB inflammatory pathway and the caspase/Bcl-2 apoptotic signaling pathway are further inhibited, thereby reducing ROS production via Nrf2-mediated mechanisms (Figure 6).

## Data Availability

The original contributions presented in the study are included in the article/Supplementary material, further inquiries can be directed to the corresponding authors.
