# Supplementary material for: Evaluation the protective role of baicalin against H2O2-driven oxidation, inflammation and apoptosis in bovine mammary epithelial cells
Source: Front Vet Sci. 2024 Dec 12;11:1504887. doi: 10.3389/fvets.2024.1504887 (PMC11669685; doi:10.3389/fvets.2024.1504887)
Supplement: Supplementary file 2 [file Data_Sheet_2.docx]

Supplementary Material

# Supplementary Data

Uncropped version of western blot protein bands

Nrf2


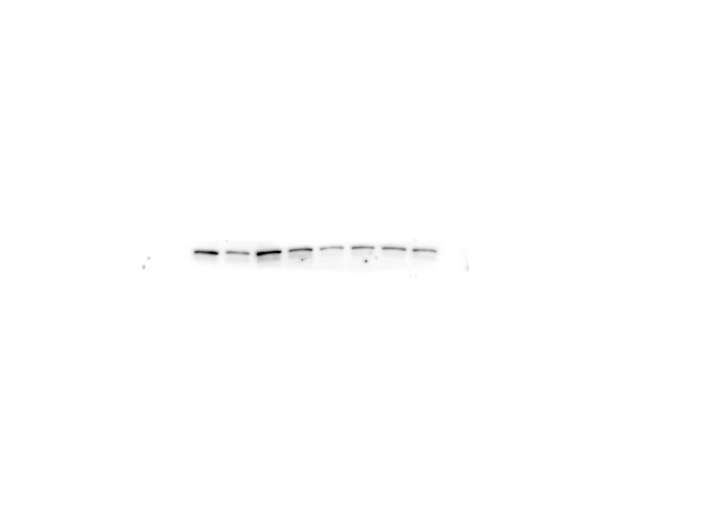

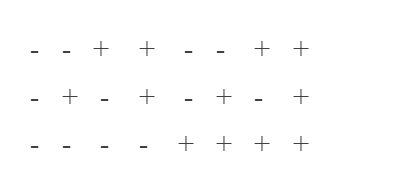


Baicalin

H2O2

RA


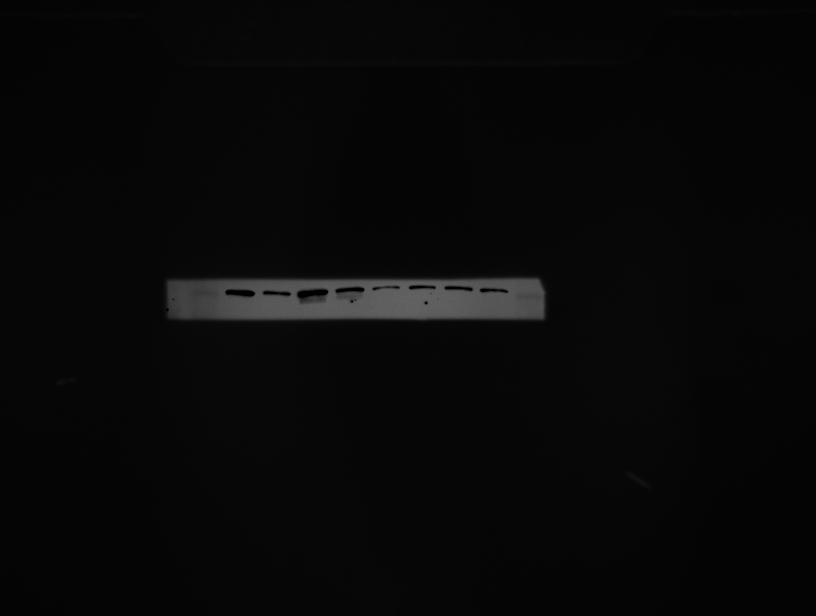


100 kDa

100 kDa

70 kDa


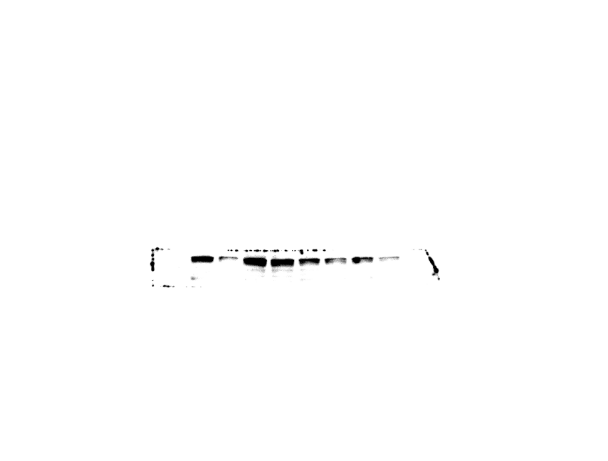


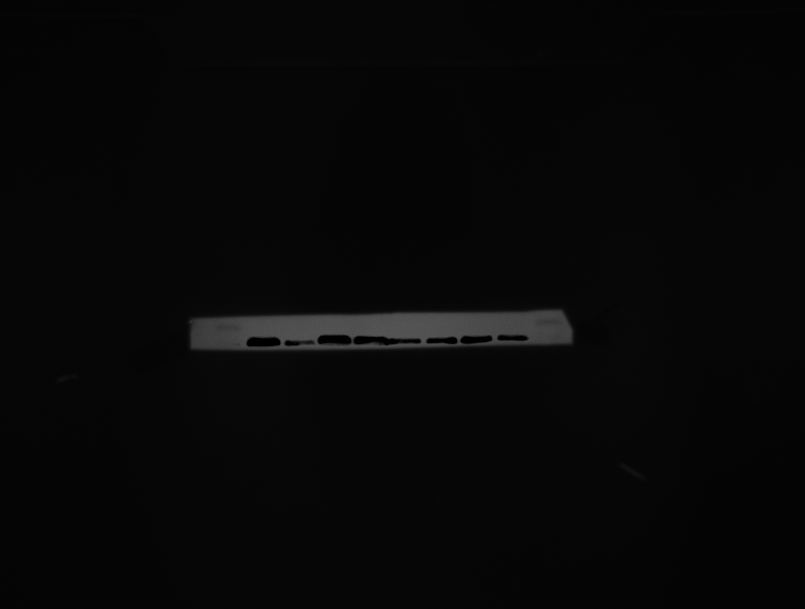


100 kDa

70 kDa

100 kDa


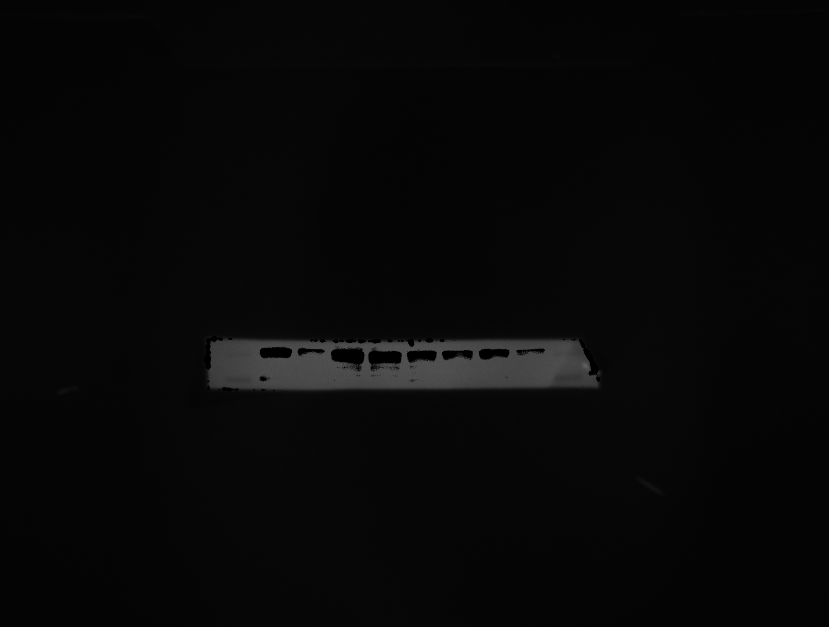


100 kDa

100 kDa

70 kDa


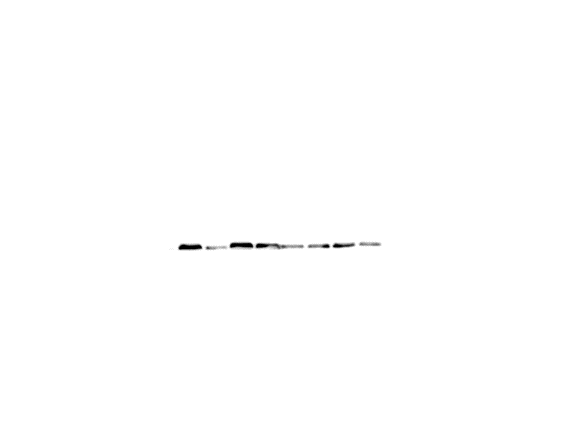


β-actin


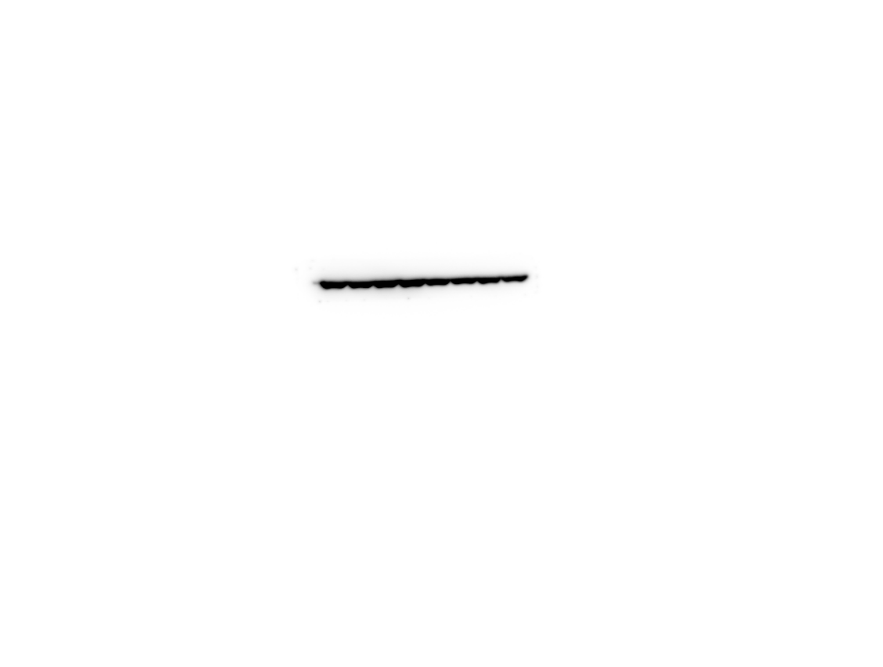

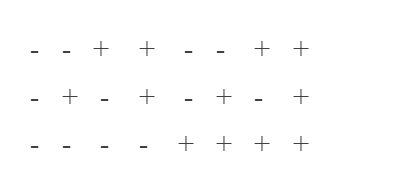


Baicalin

H2O2

RA


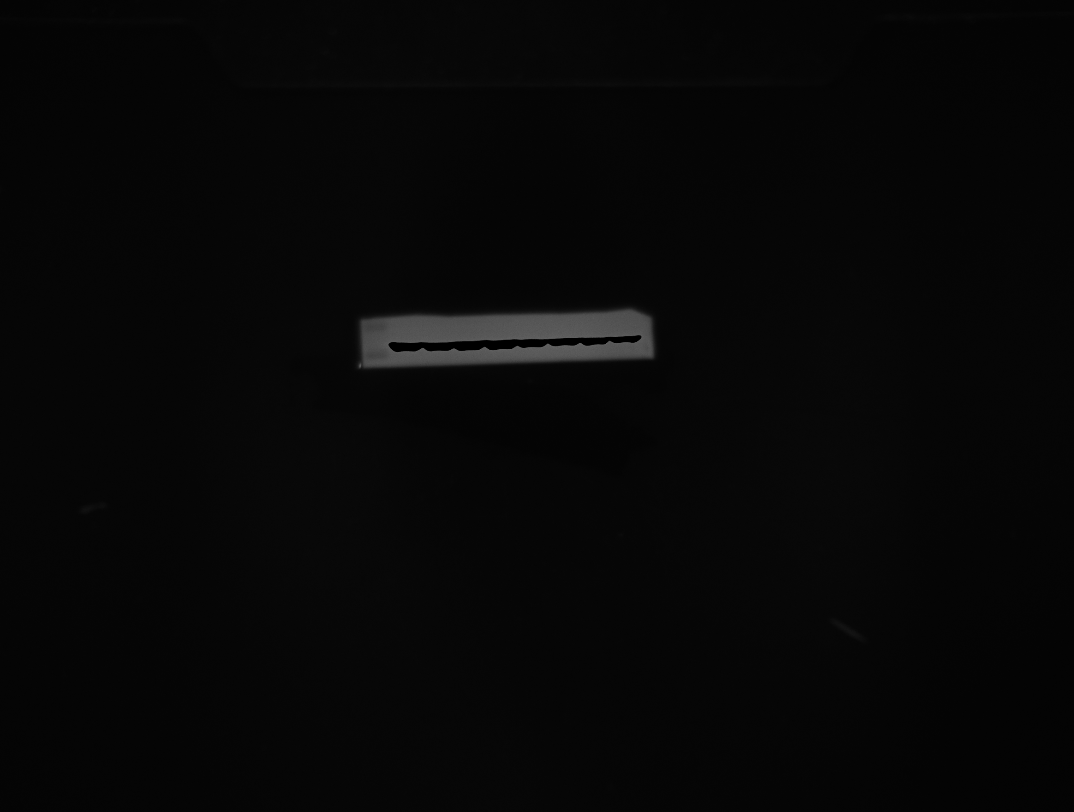


40 kDa

50 kDa

45 kDa

IκB


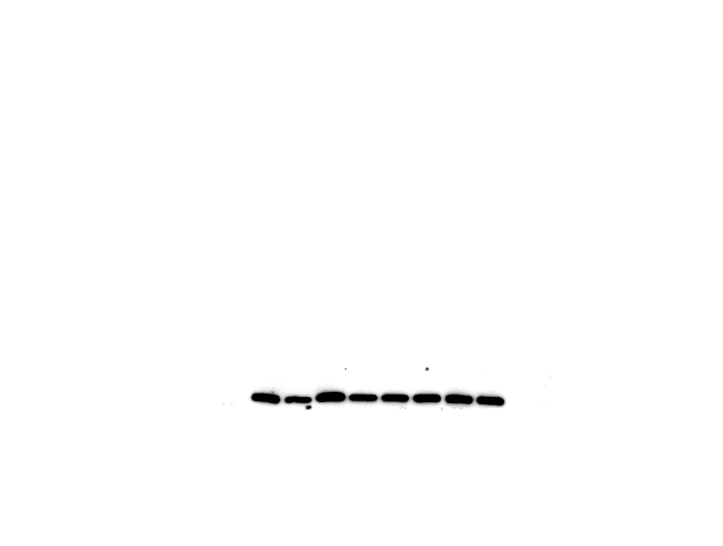

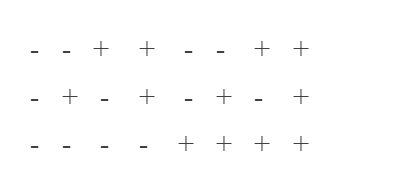


Baicalin

H2O2

RA


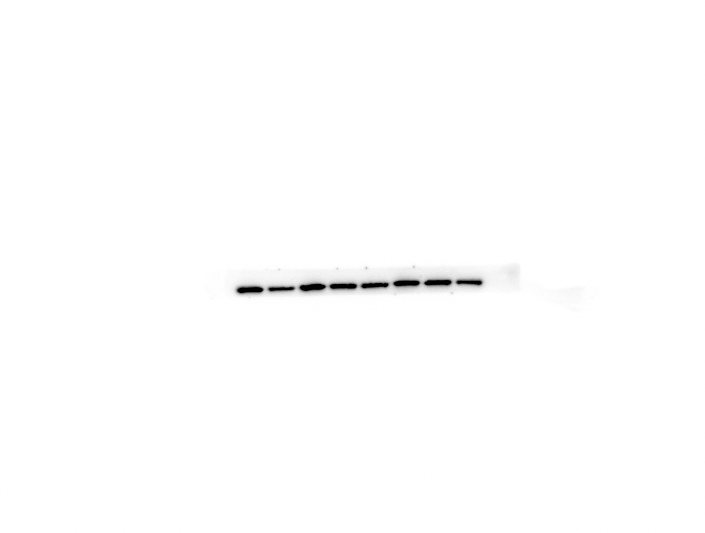

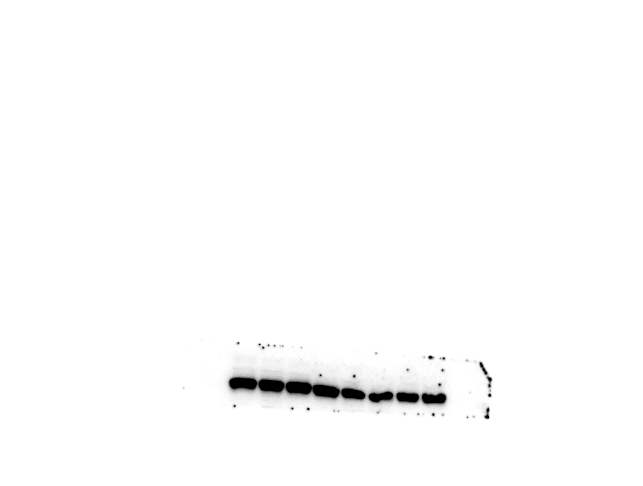


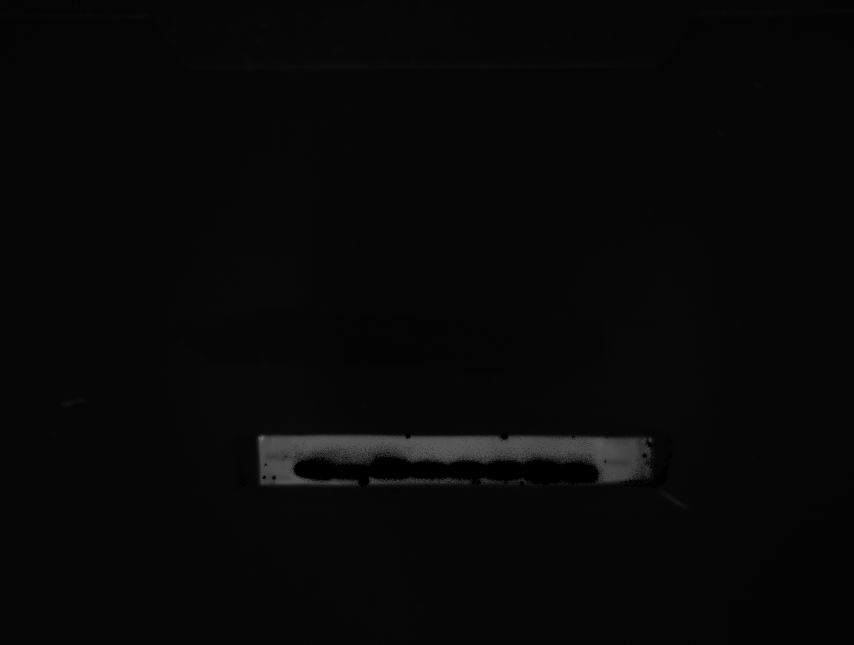


42 kDa

39 kDa

30 kDa


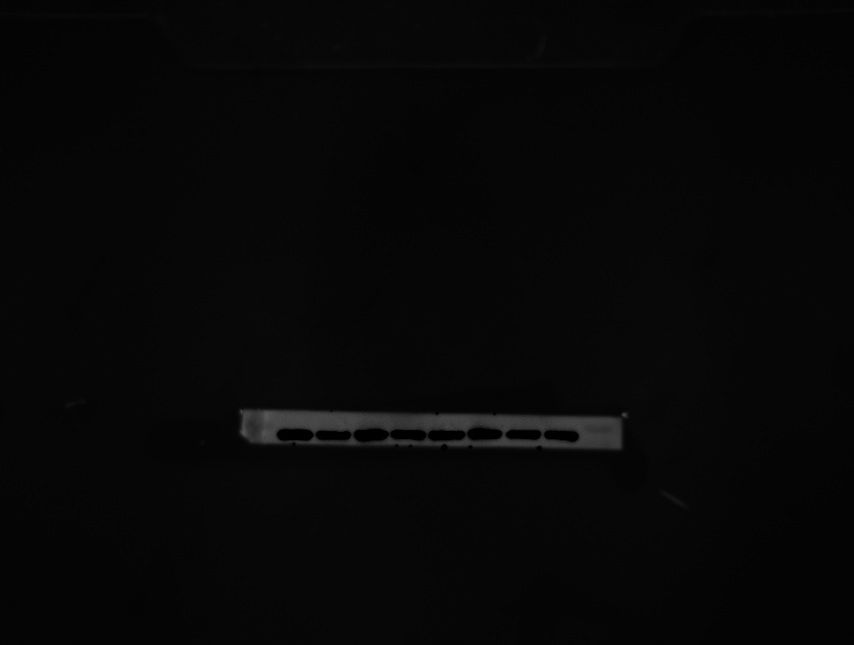


42 kDa

39 kDa


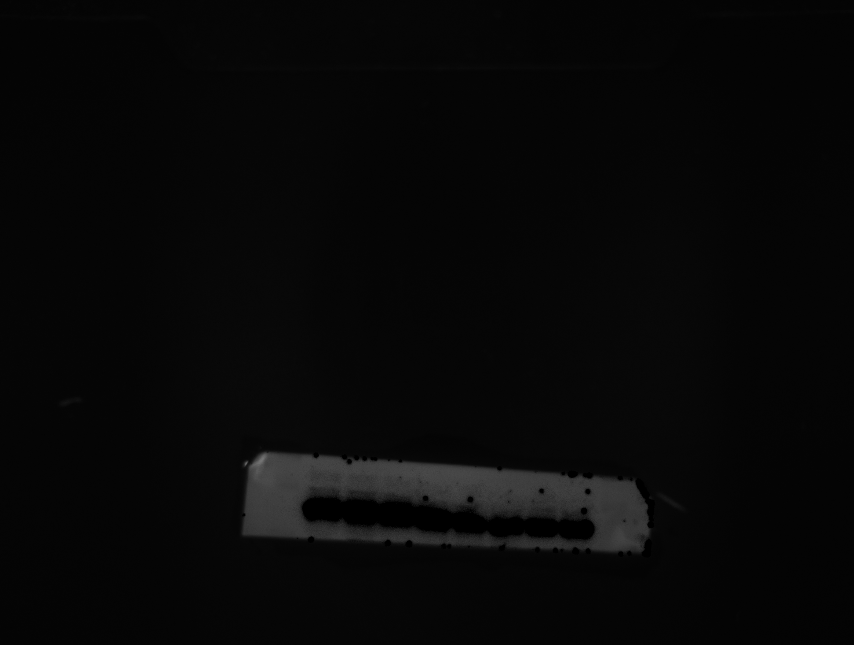


39 kDa


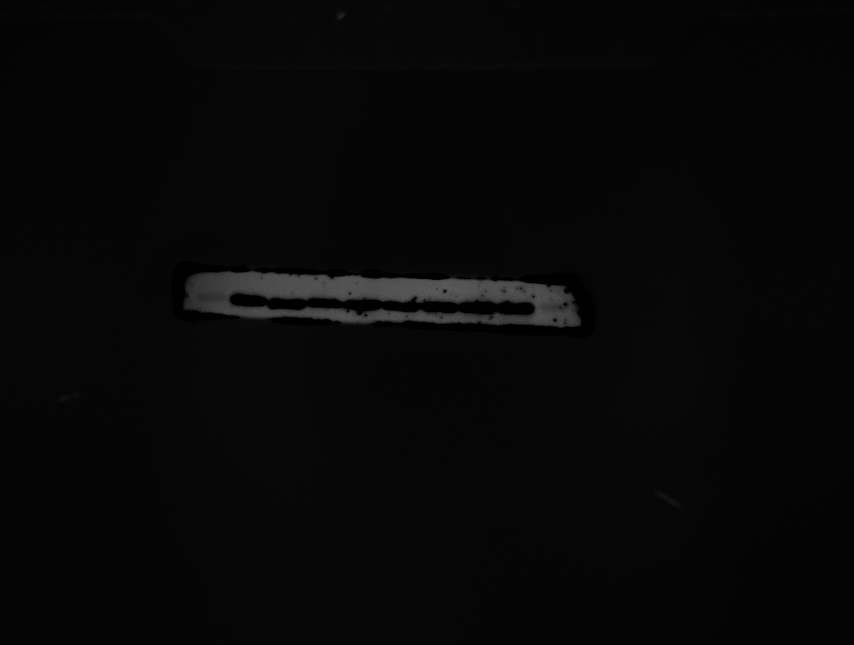

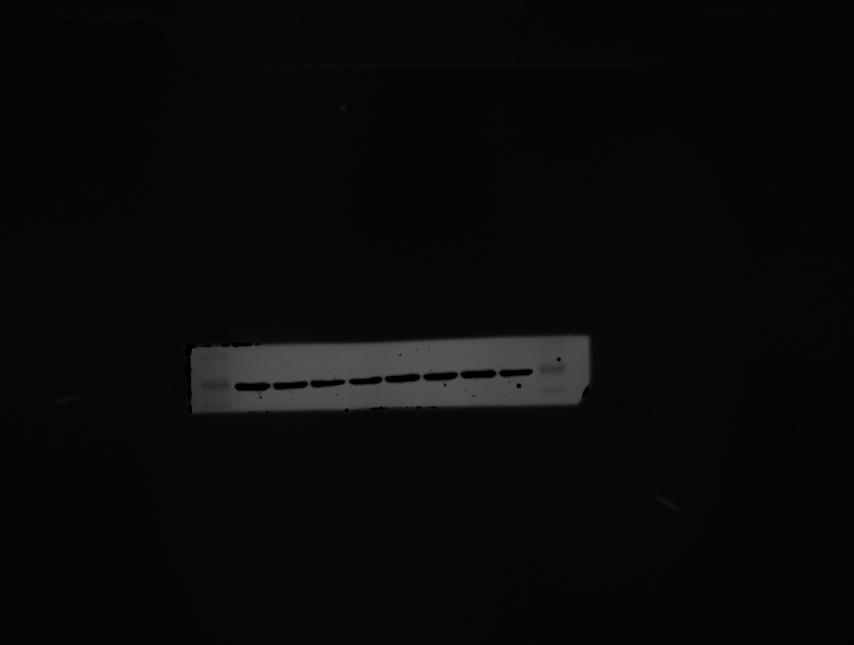

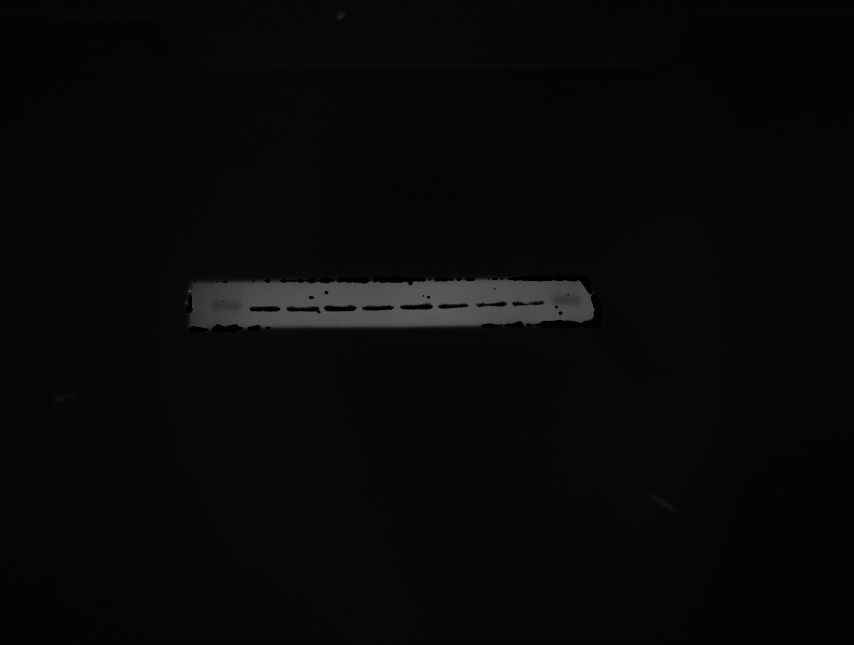

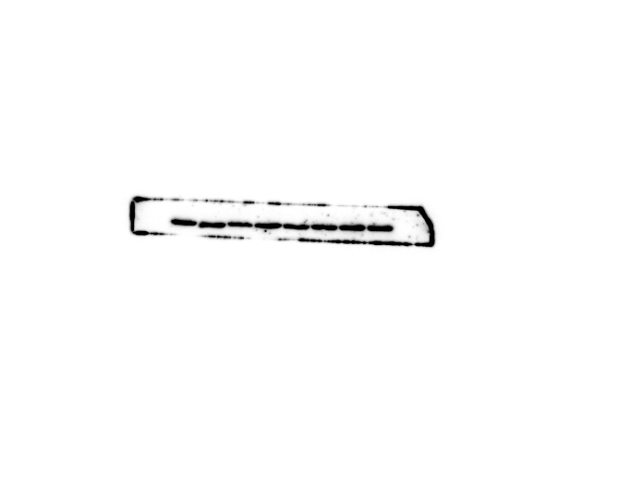

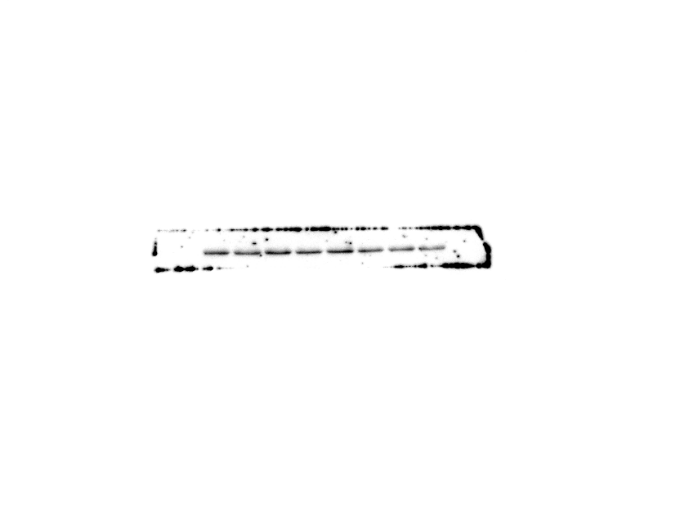

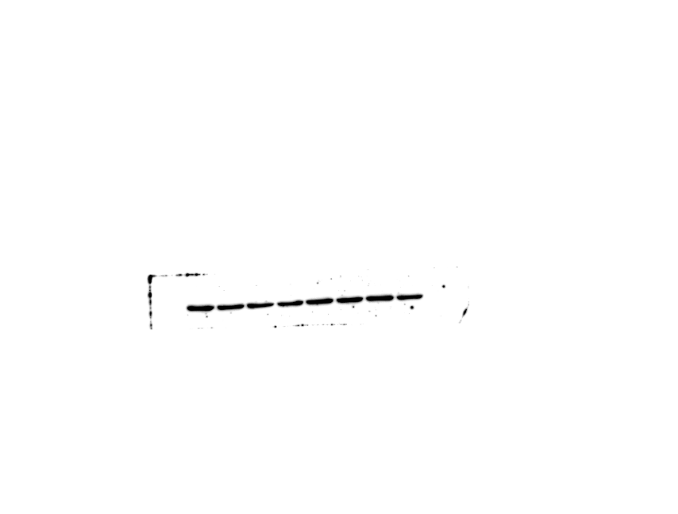

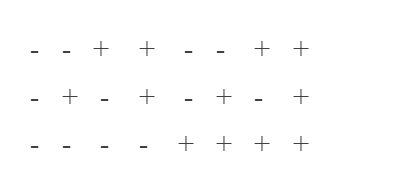
p65

110 kDa

50 kDa

70 kDa

70 kDa

70 kDa

70 kDa

70 kDa

70 kDa

p-p65


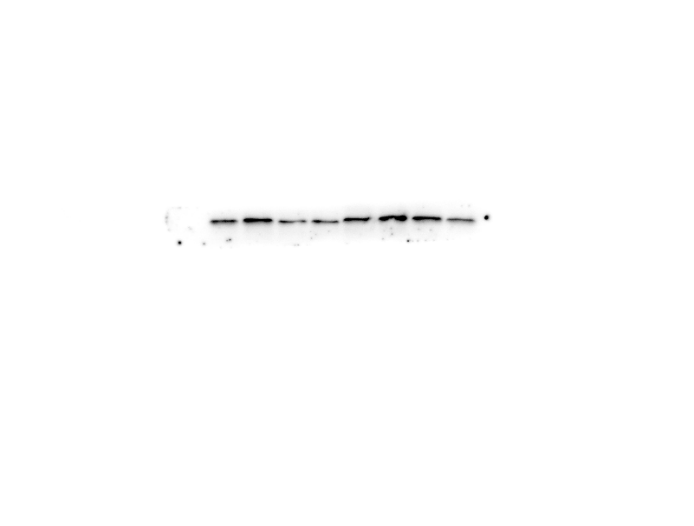

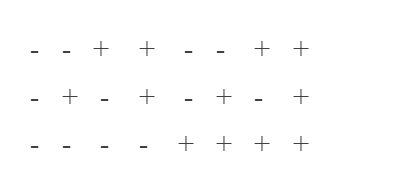


Baicalin

H2O2

RA


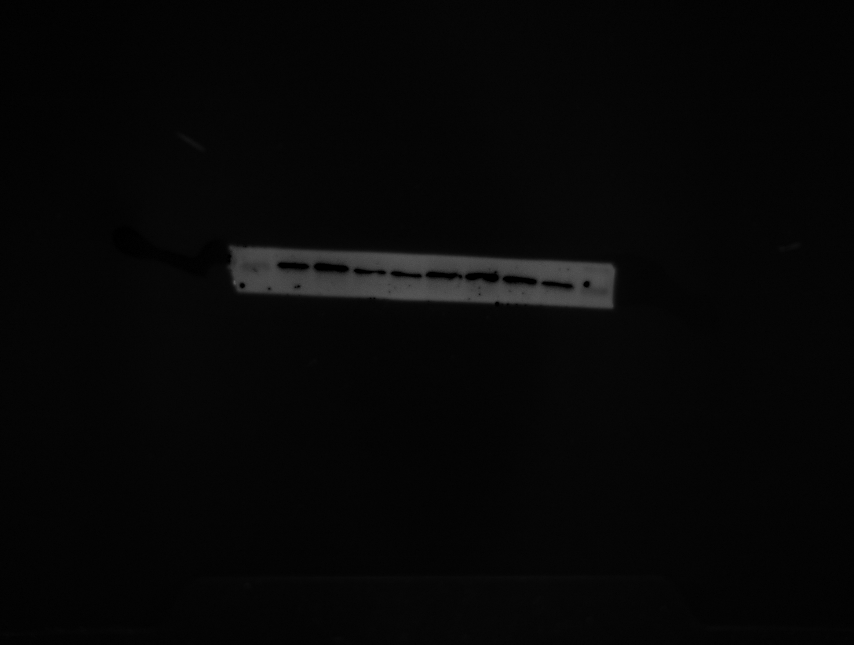


70 kDa

70 kDa


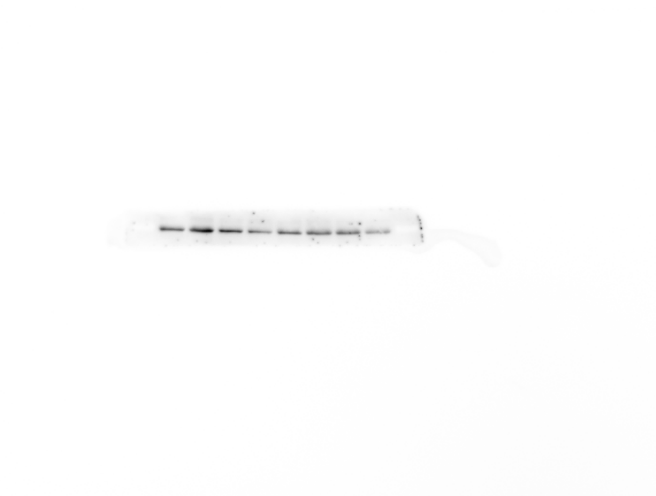

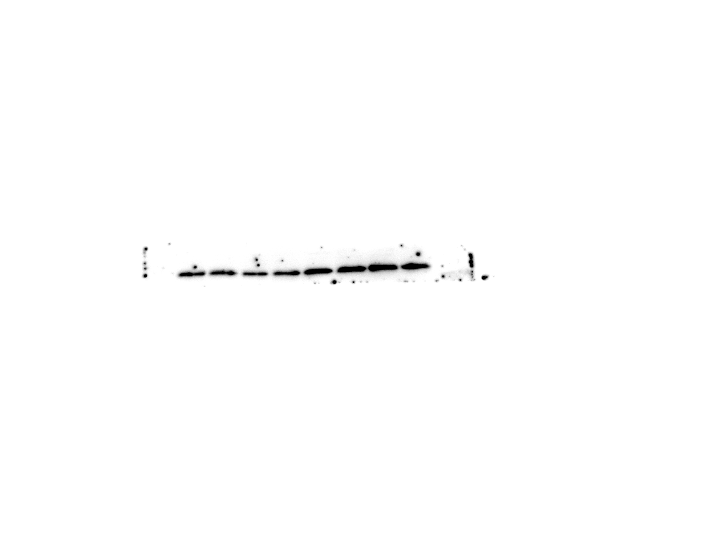


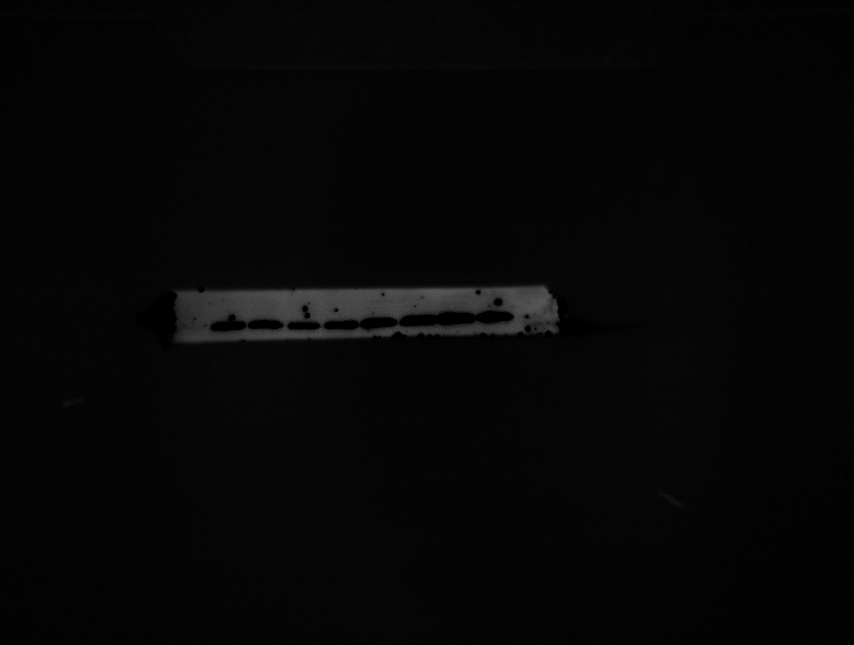


70 kDa

70 kDa


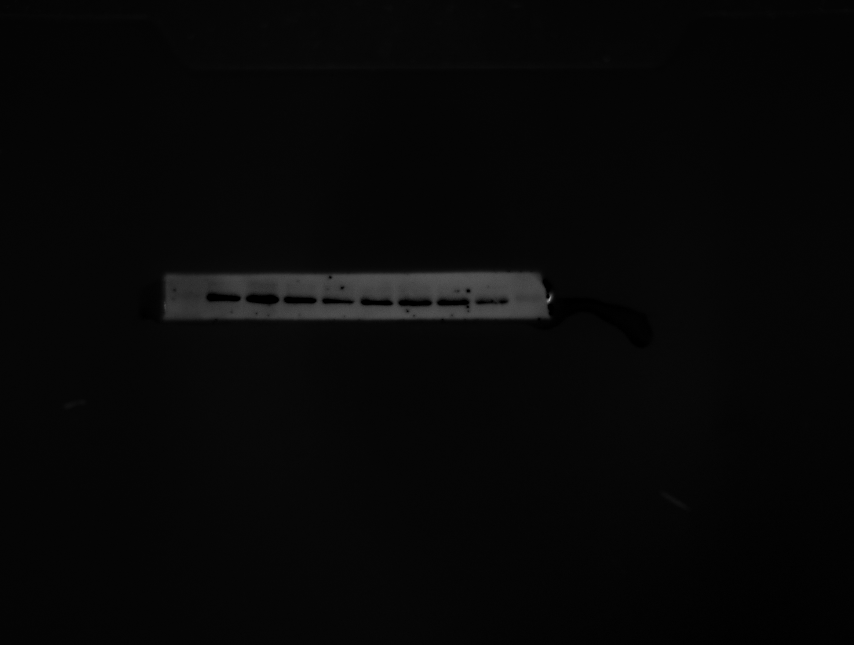


70 kDa

70 kDa

β-actin
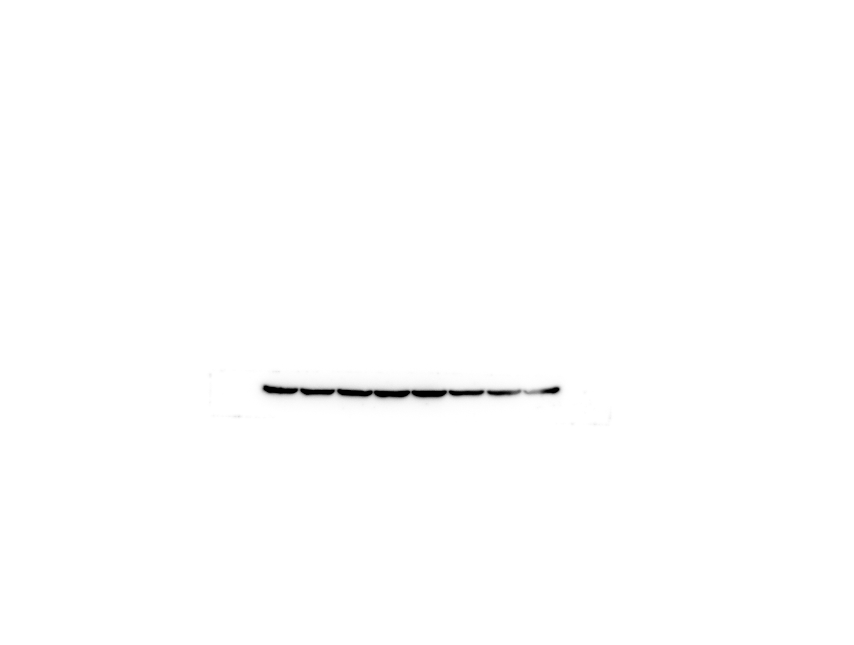

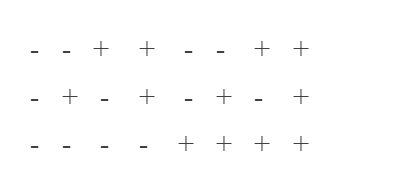


Baicalin

H2O2

RA


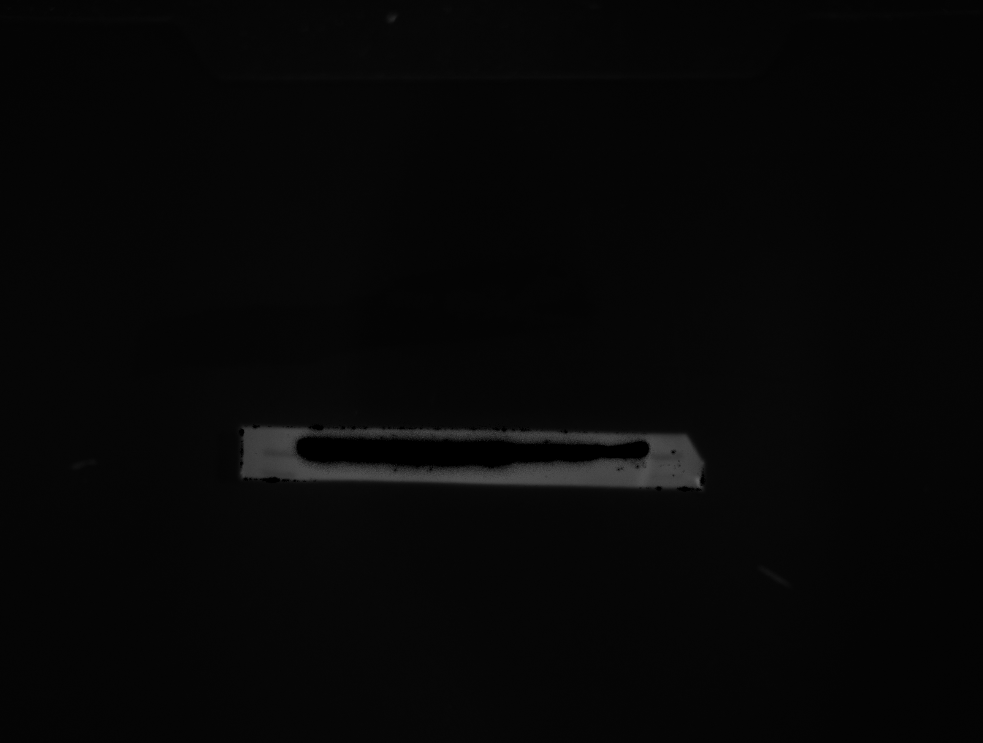


40 kDa

50 kDa

45 kDa


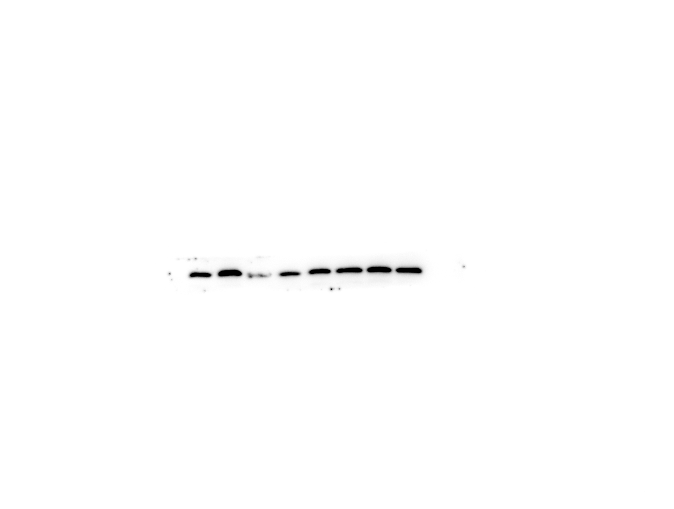

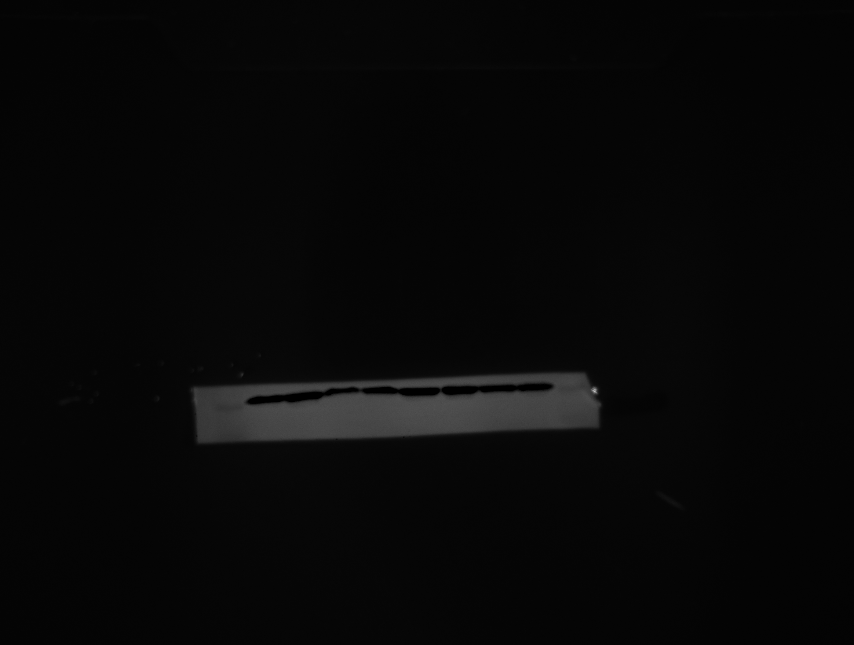

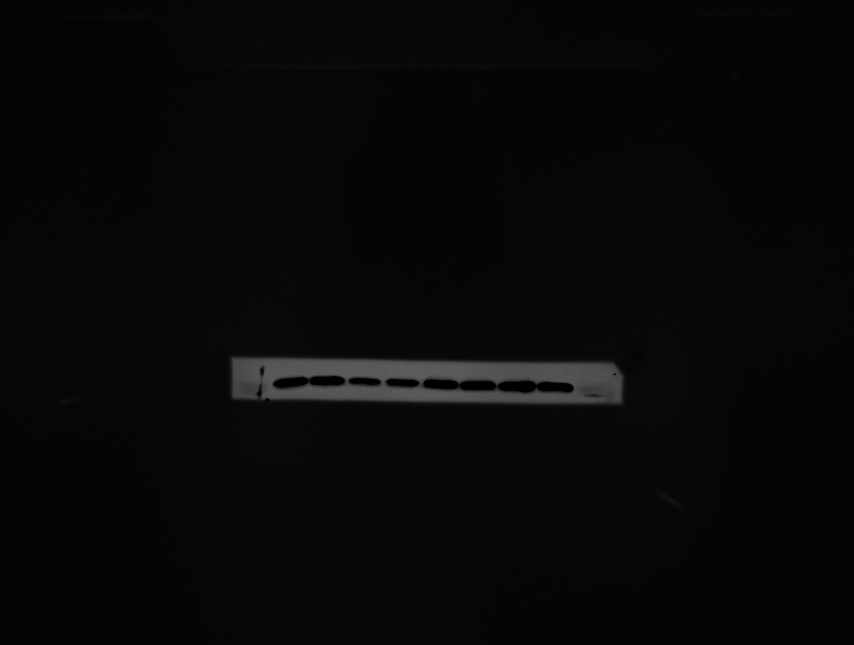

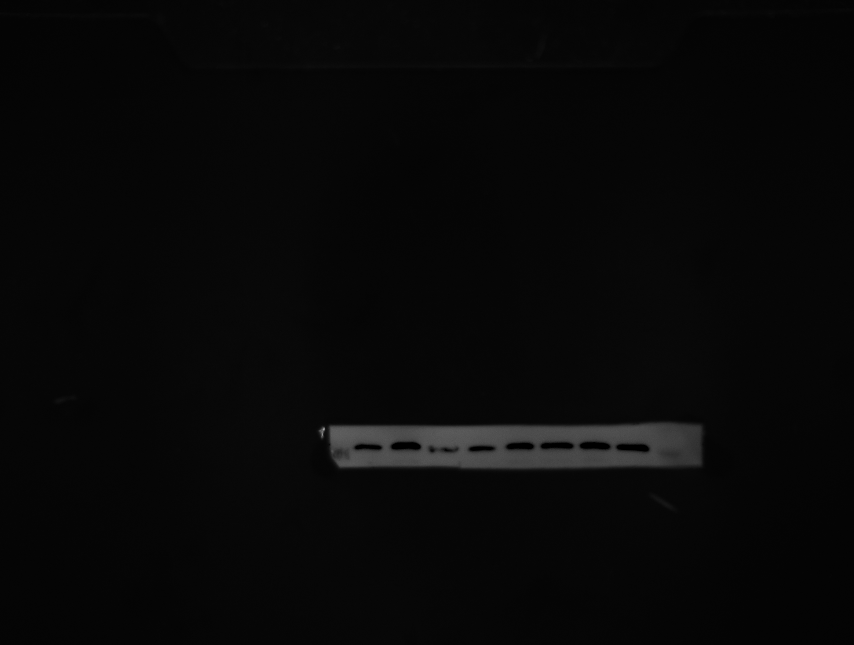

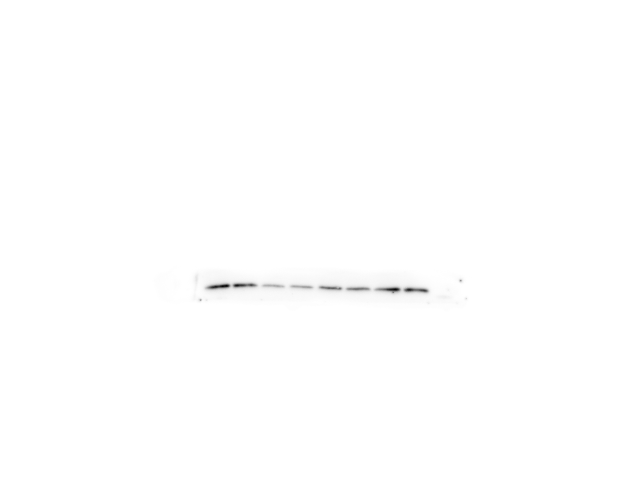

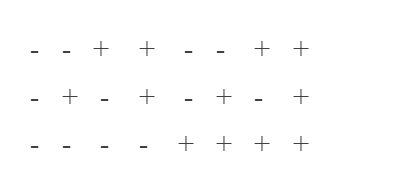

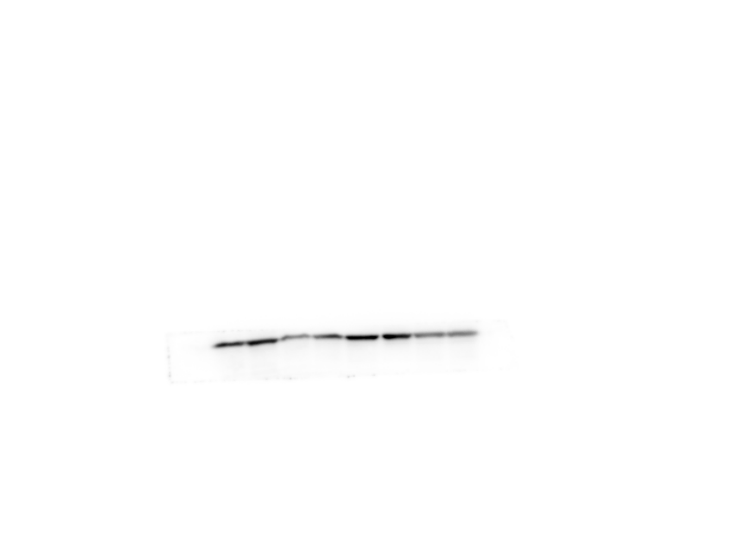
caspase3

30 kDa

30 kDa

30 kDa

Baicalin

H2O2

RA

32 kDa

32 kDa

32 kDa


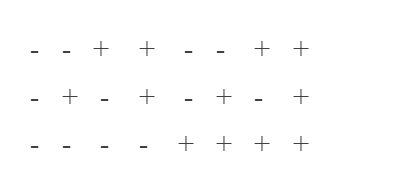

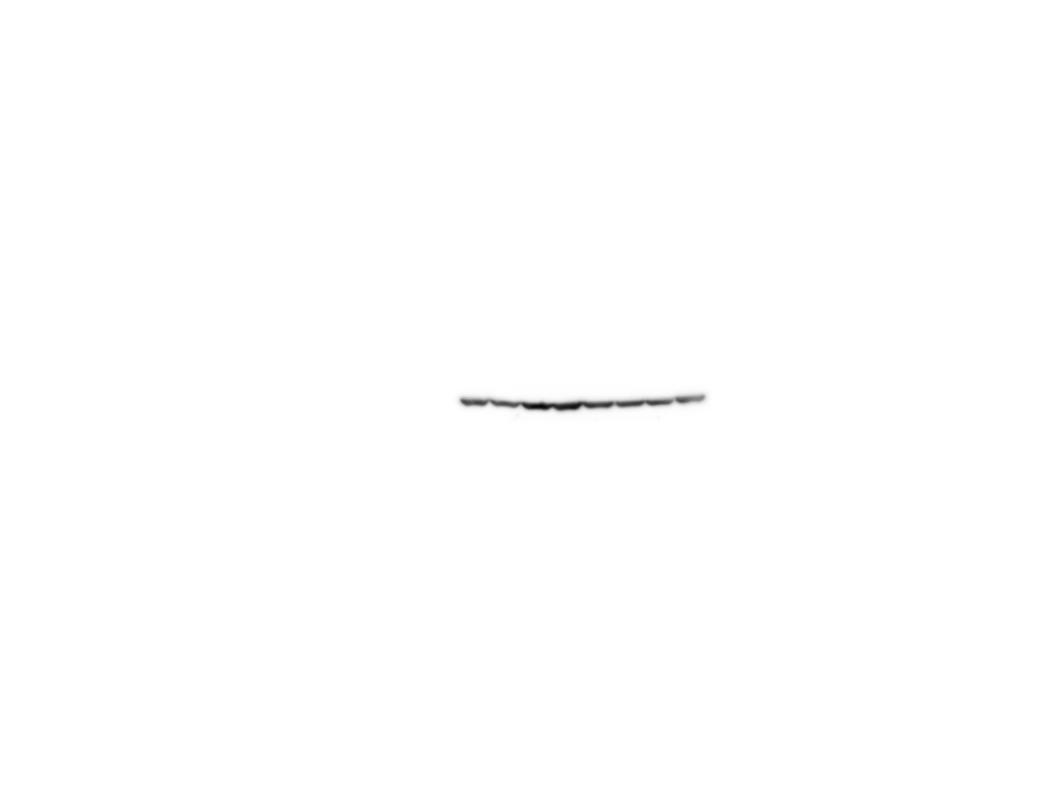
β-actin

Baicalin

H2O2

RA


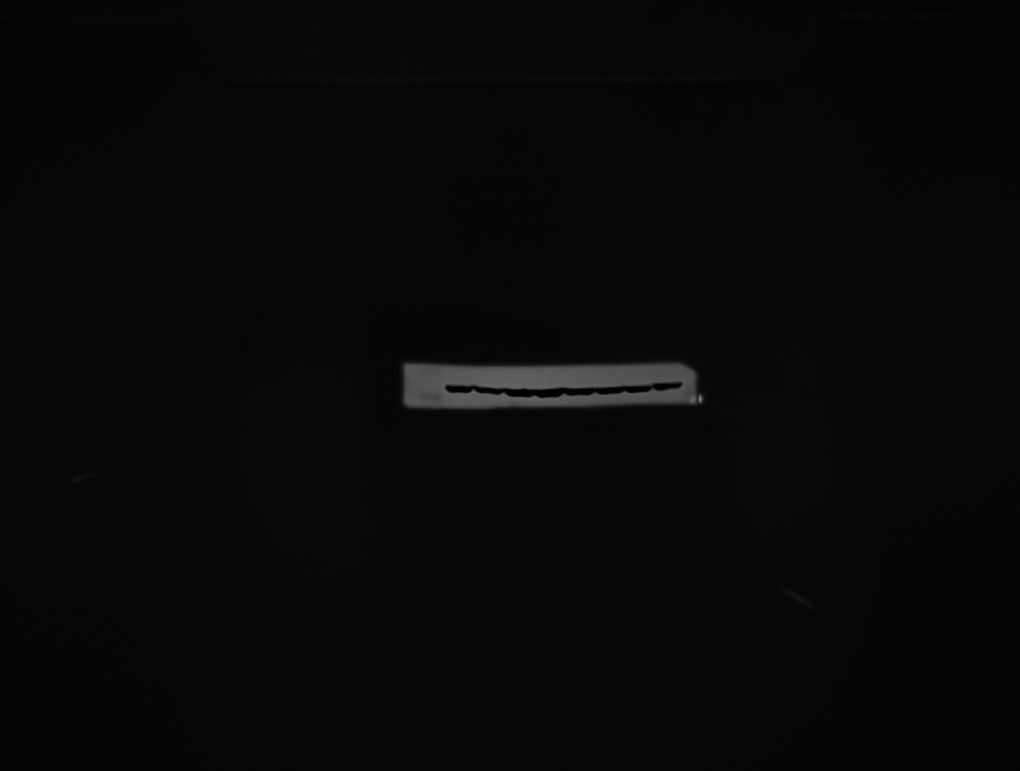


50 kDa

45 kDa
